# From Atomic Channels to Deployable Membranes: A Design-Oriented Framework for Graphene Oxide Transport, Functionalization, and Scalability

**DOI:** 10.3390/membranes16070237

**Published:** 2026-07-10

**Authors:** Awad Alzebair, Didem Aydin, İlkay Hilal Gübbük, Mustafa Ersoz

**Affiliations:** 1 Department of Biochemistry, Faculty of Sciences, Selcuk University, 42030 Konya, Türkiye; 2 Department of Chemistry, Faculty of Sciences, Selcuk University, 42030 Konya, Türkiye; didem.aydin@selcuk.edu.tr (D.A.); ilkayg@selcuk.edu.tr (İ.H.G.); mersoz@selcuk.edu.tr (M.E.)

**Keywords:** graphene oxide membranes, transport mechanisms, interlayer spacing, ion sieving, machine-learning force fields, antifouling, scalable fabrication

## Abstract

Graphene oxide (GO) membranes present a compelling alternative to the permeability-selectivity trade-off inherent in conventional polymer membranes. However, the incomplete mechanistic understanding and the absence of scalable, defect-controlled fabrication processes continue to hinder their practical deployment. This review synthesizes and integrates transport mechanisms, computational modeling, fabrication, and translational constraints across graphene-based membrane architectures into a comprehensive design-oriented framework. Five key aspects of this synthesis are highlighted. Firstly, the available evidence supports a three-regime transport model, which unifies viscous near-frictionless flow, activated molecular hopping, and solution–diffusion. This reframes selectivity as a tunable function of the C/O ratio and interlayer chemistry. Secondly, a quantitative parity analysis of literature data reveals that classical molecular dynamics tends to overestimate GO laminate water permeance by a representative factor of approximately 3–8× across the matched comparisons examined. This discrepancy can be corrected using a tortuosity–porosity factor derived from wet-state XRD. Machine-learning force fields (GAP, MACE), while still in an early stage of development with limited reported applications, narrow the residual discrepancy to within 1.5–2× in the studies reviewed. Thirdly, a tiered computational roadmap identifies nuclear quantum effects as critical for proton-transport applications but unresolved for water permeance in GO laminate geometry. Fourthly, performance across water nanofiltration, gas separation, ion recovery, and osmotic energy harvesting is benchmarked against commercial references, with explicit caveats regarding the heterogeneity of testing conditions across cited studies, alongside a technology readiness assessment. Lastly, a standardized 500-h hydraulic stability protocol is proposed to facilitate cross-laboratory comparison. Collectively, this synthesis provides a structured, albeit not exhaustively validated, basis for the discussion of next-generation membrane design.

## 1. Introduction

Membrane-based separations are fundamental to water purification, gas separation, and resource recovery [1]. However, conventional polymer membranes are limited by the inherent permeability-selectivity trade-off [2]. Graphene-based membranes offer a compelling alternative by combining atomic-scale thickness, exceptional mechanical strength, and tunable chemistry, enabling high flux without compromising selectivity [3].

Graphene is a single atomic layer of sp^2^-hybridized carbon atoms arranged in a two-dimensional honeycomb lattice [4]. Since its isolation, it has been recognized as an extraordinary material due to its high Young’s modulus, chemical stability, and intrinsic impermeability in defect-free form, making it an attractive barrier material for controlling transport at the angstrom and nanometer scales [5].

In practice, graphene oxide (GO) has proven more versatile than pristine graphene for membrane fabrication [6]. Its oxygen-containing functional groups render it hydrophilic, solution-processable, and readily amenable to scalable assembly methods, such as vacuum filtration, coating, and layer-by-layer deposition. Stacked GO laminates create sub-nanometer interlayer channels that can mediate highly selective transport of water, ions, and small molecules, making GO one of the most intensively studied two-dimensional membrane materials [7].

The field accelerated following seminal demonstrations of anomalously fast water transport and sharp ionic sieving in GO membranes, triggering broad efforts to exploit graphene-based membranes in desalination, wastewater treatment, gas separation, organic solvent nanofiltration, ion recovery, and osmotic energy harvesting. Simultaneously, these results revealed a major scientific challenge: transport in these materials is governed by a complex interplay of confinement, functionalization, swelling, defect structure, and interlayer chemistry, and the dominant mechanisms remain incompletely resolved [8].

Despite extensive reviews of graphene and GO membranes, spanning seminal transport demonstrations by Nair et al. (2012) [9] and Joshi et al. (2014) [8], fabrication advancements surveyed by Mi (2014) [10] and Wang et al. (2019) [11], and application-specific treatments, including Abraham et al. (2017) [12], the literature remains fragmented across mechanism, modeling, synthesis, and scale-up, with most existing reviews partial in scope, focusing primarily on synthesis, an isolated application area, or transport mechanisms in isolation from fabrication and scale-up constraints (see Section 1.2 below for a fuller positioning of this review relative to recent literature).

The present review brings together and synthesizes five strands of the existing literature into a single design-oriented framework; we do not claim priority over the individual underlying findings, which are drawn from and cited to prior work throughout, but rather aim to integrate them in a way not previously assembled in one place. First, we synthesize competing GO transport models into a three-regime mechanistic framework, unifying viscous flow, activated molecular hopping, and solution–diffusion, linked explicitly to GO sheet heterogeneity and C/O ratio; this reconciliation builds directly on mechanistic proposals already present in the cited primary literature. Second, we organize a tiered computational roadmap, from classical molecular dynamics through GO-specific machine-learning force fields (GAP-GO, MACE) to path-integral methods, with explicit nuclear quantum effect analysis; we note that ML force fields remain an early-stage, relatively narrowly adopted methodology for GO systems at the time of writing, and we present this roadmap as an organizing structure for an actively developing area rather than as an established practice. Third, we assemble a quantitative MD-versus-experiment parity comparison with a tortuosity correction that illustrates a representative 3–8× overprediction tendency of classical simulation in the matched studies we examined. Fourth, we propose a standardized hydraulic stability benchmark protocol intended to begin addressing the absence of cross-laboratory comparability in GO membrane durability data, a recognized but unresolved community gap. Fifth, we provide a Synthesis Route Decision Framework mapping application requirements to production methods with quantitative structural targets, presented as the authors’ design recommendation rather than an established or universally validated ranking, intended to support direct laboratory implementation as a starting point for further evaluation. Together, this synthesis aims to connect structure–transport–translation linkages in a way that complements, rather than supersedes, existing reviews of graphene-based membrane research.

Several recent reviews address GO membrane synthesis methods, transport mechanisms, or specific application areas individually, and our scope overlaps with these works in covering synthesis routes, oxidation chemistry, and transport phenomena. The distinguishing aim of the present review is not to supersede these prior treatments or to claim new primary findings, but to connect mechanism, computational modeling, fabrication scalability, and translational readiness within a single coherent narrative with consistent terminology, so that readers can trace the relationship between, for example, a synthesis decision in Section 4 and its downstream consequence for the transport mechanisms in Section 3 or the stability benchmarks in Section 7.

Despite significant progress, several barriers continue to hinder practical implementation. These include precise control of interlayer spacing, suppression of swelling under realistic operating conditions, reproducible large-area fabrication, defect minimization, and long-term chemical and mechanical stability. Reported performance values also remain challenging to compare due to their strong dependence on oxidation state, flake size, stacking order, membrane thickness, operating pressure, and feed composition. Consequently, this review critically examines graphene and graphene oxide membranes, with a focus on the structure–transport relationships, fabrication strategies, functionalization routes, computational modeling, and application-specific constraints. Rather than merely cataloging studies in isolation, we extract design principles that elucidate how permeability, selectivity, and stability can be modulated through controlled manipulation of membrane chemistry and architecture. By organizing the literature around mechanistic and translational questions, we endeavor to provide a framework for the design and deployment of next-generation graphene-based membranes.

### 1.1. Scope and Literature Search Methodology

Literature was identified through systematic searches of Web of Science, Scopus, and Google Scholar using terms, including “graphene membrane,” “graphene oxide membrane,” “GO laminate transport,” “nanoporous graphene separation,” and “reduced graphene oxide membrane,” supplemented by application-specific terms (“desalination,” “gas separation,” “ion sieving,” “osmotic energy harvesting”). The search spans 2004–2024, with an emphasis on post-2013 work coinciding with the emergence of GO laminate membranes as a dominant research focus. Studies were included if they reported original experimental transport data, directly relevant computational results, or critical mechanistic analysis; they were excluded if graphene functioned solely as an additive or if experimental conditions were insufficiently reported for meaningful comparison. Review articles were included selectively where they provided a synthesis or benchmarking unavailable in primary sources. Approximately 150 primary sources are drawn upon, selected for their mechanistic, fabrication, or application relevance and their capacity to yield design implications beyond their immediate experimental context.

The approximately 150 primary sources cited in this review represent a critically curated, analytically selected subset rather than an exhaustive retrieval. This review does not adhere to a formal PRISMA protocol, which is designed for systematic exhaustive retrieval to support clinical meta-analysis. The present work is a design-oriented, narrative–analytical review whose objective is mechanistic synthesis and the extraction of design principles. A PRISMA flow diagram would imply an exhaustive-retrieval mandate, which is inconsistent with this analytical intent.

### 1.2. Reader’s Guide

Readers may navigate this review selectively according to their primary interest. Experimentalists will find the most directly applicable material in Section 2, Section 2.1 and Section 2.2 (GO structural characterization), Section 5 (functionalization and interlayer spacing control), Section 6.5 (fouling mechanisms), and Section 7.3 (stability benchmarking). Computational modelers should prioritize Section 3.3 andSection 3.4, which develop the tiered simulation roadmap, the MD parity framework, and the continuum parameterization protocol. Process engineers and translational researchers are directed to Section 4 (fabrication strategies), Section 6 (application benchmarking), Section 7.4 and Section 7.5 (economic feasibility and TRL assessment), and the five design recommendations in Section 8. All readers are encouraged to consult Table 1 (synthesis route decision framework) and Table 6 (TRL assessment) as standing reference points.

## 2. Structural and Physicochemical Properties of Graphene and Graphene Oxide

### 2.1. Graphene: Structure and Synthesis

Pristine graphene is composed of a single atomic layer of carbon atoms arranged in a hexagonal lattice [13,14], characterized by a carbon-carbon bond length of 1.42 Å. Each carbon atom engages in three in-plane σ-bonds with its nearest neighbors, while the remaining out-of-plane p-orbital electrons collectively form a delocalized π-electron system that extends across the entire basal plane [15]. It is this π-conjugation that underlies graphene’s celebrated electronic properties, including its exceptionally high carrier mobility and ambipolar field-effect behavior. However, for membrane applications, it is the material’s mechanical and chemical characteristics that assume primary significance [16]. The in-plane tensile strength (~130 GPa) and Young’s modulus (~1 TPa) establish graphene as the stiffest material known [17], endowing it with the capacity to sustain substantial pressure differentials without mechanical failure, a prerequisite for any pressure-driven separation operation. Equally relevant is graphene’s intrinsic impermeability: the electron density of the aromatic ring is sufficiently high to repel even the smallest gas molecules, including helium, rendering defect-free graphene intrinsically impermeable to all molecular transport, a property that necessitates deliberate modification for membrane applications [18]. This impermeability, paradoxically, is both graphene’s most attractive and most challenging feature for membrane design. It necessitates the deliberate introduction of nanoscale pores to enable selective permeation while preserving the structural integrity of the lattice [19].

The crystallographic perfection of graphene also has direct implications for its chemical reactivity [20]. The basal plane of pristine graphene is chemically inert under ambient conditions, a consequence of the thermodynamic stability conferred by full sp^2^ hybridization and π-delocalization. Functionalization of the basal plane, therefore, requires either covalent disruption of the aromatic lattice, which introduces sp^3^ defect sites and locally degrades mechanical properties, or non-covalent adsorption strategies that preserve the carbon framework [20]. Edge sites and lattice defects, by contrast, are considerably more reactive and serve as preferential anchoring points for chemical modification. This distinction between basal plane and edge reactivity is of practical importance in the design of functionalized graphene membranes, where the spatial distribution of chemical groups governs both transport selectivity and membrane stability [21].

Several distinct synthesis routes have been developed for the production of graphene intended for membrane applications, each carrying its own set of practical trade-offs [22]. Chemical vapor deposition (CVD) on metal substrates, most commonly copper or nickel foils, remains the most widely adopted method for obtaining large-area monolayers of comparatively high crystalline quality [23,24]. In this process, carbon-containing precursor gases, such as methane, are thermally decomposed at elevated temperatures, typically between 900 °C and 1050 °C, and the resulting carbon species migrate across the metal surface to nucleate and grow a continuous graphene film. The choice of substrate metal exerts a significant influence on the outcome: copper, owing to its low carbon solubility, favors the self-limiting growth of monolayer graphene, whereas nickel, with its higher carbon solubility, tends to yield multilayer films through a precipitation mechanism [25]. Despite the high quality of CVD-grown graphene, the subsequent transfer of these films onto non-conductive substrates remains an inherently imperfect process. Conventional wet-transfer protocols, in which a polymeric support layer, most commonly poly(methyl methacrylate) (PMMA), is deposited onto the graphene surface prior to copper etching, consistently introduce wrinkles, tears, and grain boundaries that function as preferential, non-selective transport pathways in the final membrane. The practical severity of this problem is reflected in reported defect densities: standard PMMA-assisted wet transfer yields defect densities in the range of 10^10^–10^12^ cm^−2^ [26], whereas electrochemical delamination methods, which avoid substrate etching entirely by exploiting the electrochemical reduction in water at the graphene–copper interface to separate the film without chemical dissolution of the metal, reduce this figure by two to three orders of magnitude, to approximately 10^8^–10^9^ cm^−2^. Even at the lower end of the PMMA-transfer range, a defect density of 10^10^ cm^−2^ corresponds to a mean inter-defect spacing of roughly 100 nm, comparable to or smaller than the lateral dimensions of many practical nanoporous graphene membrane apertures, meaning that transfer-induced defects can dominate transport behavior and entirely mask the selectivity of deliberately engineered nanopores [27]. These considerations provide the primary motivation for the growing body of literature on direct, polymer-free, and electrochemical transfer methodologies, which collectively aim to decouple the defect penalty of the transfer step from the intrinsic structural quality of the CVD-grown film.

Mechanical exfoliation, commonly known as the “Scotch tape” method, and the technique employed by Novoselov and Geim to isolate graphene [28], yields pristine single-crystal monolayer flakes ideally suited for fundamental studies, but is fundamentally incompatible with large-scale production (flake dimensions restricted to tens of micrometers, low yield, no automation pathway). Consequently, mechanically exfoliated graphene serves primarily as a laboratory model material rather than a practical membrane feedstock, and is not discussed further here [29].

Liquid-phase exfoliation (LPE) of graphite in appropriate solvents presents a more scalable alternative, producing graphene flakes that are dispersible in solution and thus directly amenable to thin-film and composite fabrication strategies [30,31]. The underlying principle involves matching the surface energy of the solvent to that of graphene, thereby minimizing the energetic penalty associated with separating individual layers from the graphite stack. Solvents, such as N-methyl-2-pyrrolidone (NMP) and dimethylformamide (DMF), have been identified as particularly effective [30], although aqueous surfactant-assisted exfoliation has gained traction as a less toxic alternative [32]. The principal drawbacks of LPE are a reduction in lateral flake dimensions compared with CVD-grown or mechanically exfoliated graphene, and a tendency toward incomplete exfoliation, resulting in the co-production of few-layer and multilayer species alongside genuine monolayers. These morphological heterogeneities can complicate the reproducible fabrication of membranes with well-defined transport properties, and post-exfoliation sorting strategies, including density gradient ultracentrifugation, have been explored as means of isolating monolayer-enriched fractions.

Epitaxial growth on silicon carbide (SiC) substrates through high-temperature thermal decomposition presents a noteworthy synthesis route, particularly in the realm of electronic device applications [33]. While this approach yields graphene with high structural uniformity and eliminates the transfer step inherent in Chemical Vapor Deposition (CVD) processes, the necessity for single-crystal SiC wafers and ultra-high vacuum conditions renders it prohibitively expensive for membrane fabrication at any practical scale. Consequently, this synthesis method is mentioned primarily for completeness, as its contribution to the membrane literature has been limited thus far. Collectively, the diversity of available synthesis methods reflects both the breadth of graphene research and the ongoing absence of a single production route that simultaneously fulfills the requirements of high structural quality, large-area uniformity, and cost-effective scalability, a challenge that remains central to the field’s translational aspirations [34].

Table 1 summarizes the practical implications of these trade-offs for membrane design. It maps key application requirements, structural quality, scalability, and separation performance targets to the optimal synthesis route for each.

The BEST/MODERATE/POOR ratings and target values presented in Table 1 reflect the authors’ synthesis of reported structural quality (C/O ratio control, defect density, d-spacing uniformity), scalability (demonstrated maximum membrane area, throughput), and separation performance (permeance and rejection ranges) for each synthesis route, drawn from the specific studies cited in the corresponding rows. These classifications are not the output of a quantitative scoring algorithm or a systematic survey of community opinion, but rather a structured design recommendation intended to help readers navigate trade-offs between competing synthesis routes. Readers with different application priorities, access to different starting materials, or emphasis on criteria not captured here may reasonably reach different conclusions; the ratings should therefore be treated as a starting point for synthesis route selection rather than a definitive or universally validated verdict.

**Table 1:** Graphene Synthesis Route Decision Framework: Mapping Application Requirements to Optimal Production Method.

Application Requirement	CVD (Cu foil)	Hummers GO	Tour GO	Liquid-Phase Exfoliation	Decision Rationale & Key Metrics
A. Structural Quality & Defect Density					
Minimum defect density required (e.g., nanoporous single-layer membrane)	✓ BEST 10^8^–10^9^ cm^−2^ (best-case; typical 10^9^–10^10^ cm^−2^) (electrochemical delamination) [35,36]	✗ POOR Inherently defective basal plane [37] C/O 1.3–1.8	○ MODERATE Higher sp^2^ retention; C/O 2.1–2.6	✗ POOR Multilayer stacking; edge defects dominant	Use CVD + electrochemical delamination. Target: defect density ≤ 10^9^ cm^−2^ (best-case); I(D)/I(G) < 0.1 by Raman. [35]
Laminate with controllable d-spacing (e.g., ion sieving)	✗ NOT APPLICABLE Single-layer; no laminate d-spacing	**○** GOOD d-spacing 0.8–1.2 nm (wet); wide tuning range but swelling risk	✓ BEST d-spacing 0.65–0.98 nm (cross-linked); narrower distribution [12]	○ MODERATE Broad flake-size distribution limits d-spacing uniformity	Prefer Tour GO + epoxy cross-linking. Target d-spacing: 0.65–0.98 nm (XRD); C/O ≥ 2.1 before cross-linking. FWHM < 0.05 nm [12]
B. Scalability & Manufacturing Readiness					
Large-area (m^2^-scale) membrane production	○ EMERGING R2R CVD demonstrated at 30-inch roll width [38]	✓ BEST Slot-die/vacuum filtration; batch yields > 10 g per synthesis	✓ BEST Lower defect density than Hummers; comparable throughput	○ MODERATE Scalable but flake-size polydispersity requires centrifugation	Hummers or Tour GO for immediate scale-up. Key metric: membrane yield (m^2^ per kg GO precursor). Target ≥ 50 m^2^ kg^−1^ (pilot-scale estimate). [39,40]
C. Separation Performance Targets					
Monovalent/divalent ion selectivity (desalination, ion recovery)	✗ POOR No intrinsic charge selectivity in pristine graphene	○ MODERATE High swelling risk at d-spacing > 1.0 nm reduces selectivity	✓ BEST Stable d-spacing 0.65–0.98 nm; Na^+^/Mg^2+^ selectivity > 100 reported [12]	✗ POOR Polydisperse flakes create bypass channels	Tour GO + epoxy cross-linking. Target: d-spacing ≤ 0.72 nm for divalent exclusion; XRD FWHM < 0.05 nm [12]
Gas separation (H_2_/CO_2_, He/CH_4_)	✓ BEST Atomic thickness; sub-nm pores engineered by ion bombardment/UV-ozone [41,42]	✗ POOR Functional groups cause water co-adsorption, blocking gas channels	○ MODERATE rGO (C/O > 4) post-reduction improves gas selectivity	✗ POOR Multilayer stacking eliminates gas permeance advantage	CVD graphene with controlled nanoporation. Target: pore density 10^12^ cm^−2^ [41] H_2_/CO_2_ selectivity ≥ 10 (Knudsen limit ×4.7)
Organic solvent nanofiltration (OSN) (MWCO 200–1000 Da)	✗ POOR Solvent instability of transfer films; delamination in organic media	○ MODERATE Swelling in polar aprotic solvents (DMF, NMP) limits stability	✓ BEST Higher sp^2^ content reduces solvent swelling; C/O 2.1–2.6 preferred	○ MODERATE Feasible for non-polar solvents; MWCO control limited by polydispersity	Tour GO + diamine cross-linking for polar solvents. Target: permeance ≥ 5 L m^−2^ h^−1^ bar^−1^ in DMF; Rose Bengal rejection ≥ 95% [43,44]

**Legend:** ✓ BEST = recommended first choice; ○ MODERATE = viable with additional processing steps; ✗ POOR/NOT APPLICABLE = not recommended for this requirement. Citation notes on quantitative thresholds: Defect density (CVD + electrochemical delamination): ≤10^9^ cm^−2^ represents best-case laboratory conditions; typical independent-laboratory values fall within 10^9^–10^10^ cm^−2^, and the lower bound of 10^8^ cm^−2^ has not yet been broadly reproduced. See [11,45]. d-spacing FWHM < 0.05 nm (Tour GO): Derived from XRD lineshape analysis. See [12]. Membrane yield ≥ 50 m^2^ kg^−1^: Pilot-scale estimate; values from individual laboratory preparations vary widely. See [39,40]. I(D)/I(G) < 0.1 target for CVD: [45,46]. *Pore density 10^12^ cm^−2^ for CVD nanoporous graphene*: [41,42]. OSN permeance ≥ 5 L m^−2^ h^−1^ bar^−1^ in DMF, Rose Bengal rejection ≥ 95%: [43,44]. Practitioners should treat all thresholds as targets informed by current best practice rather than universally reproducible specifications. Abbreviations: CVD, chemical vapor deposition; GO, graphene oxide; R2R, roll-to-roll; rGO, reduced graphene oxide; MWCO, molecular weight cut-off; FWHM, full width at half maximum; DMF, dimethylformamide; XRD, X-ray diffraction; LPE, liquid-phase exfoliation.

### 2.2. Graphene Oxide: Chemical Structure and Properties

Graphene oxide (GO) is predominantly synthesized through the oxidation of graphite using robust oxidizing agents [47]. The Hummers method and its variants are the most prevalent approaches, although the Tour method, which employs phosphoric acid as a co-oxidant under milder conditions, has gained significant adoption due to its improved structural regularity and reduced toxic byproduct generation. The resulting GO sheets exhibit a heterogeneous distribution of oxygen functional groups [48], as described by the Lerf–Klinowski model [49]: epoxide and hydroxyl groups are predominantly located on the basal plane, while carboxyl and carbonyl groups are concentrated at the sheet edges. The extent of oxidation, and consequently the density and distribution of these functional groups, is significantly influenced by synthesis conditions and profoundly affects the resulting membrane properties [50].

The presence of oxygen-containing groups disrupts the sp^2^ conjugation of the graphene lattice, introducing sp^3^-hybridized carbon centers and increasing the interlayer d-spacing to typically 0.7–1.2 nm under humid conditions, compared to 0.335 nm for pristine graphite [51]. This expanded interlayer spacing, coupled with the corrugated, amphiphilic character of GO sheets wherein hydrophobic graphitic domains and hydrophilic oxygenated regions coexist within the same basal plane, serves as the structural foundation for selective transport channels in GO laminates. The ionizable carboxylate groups at sheet edges provide a pH-dependent electrostatic surface charge that plays a pivotal role in Donnan exclusion-based ion rejection [52].

A critical parameter in membrane design is the carbon-to-oxygen (C/O) ratio, which exhibits significant variation depending on the synthesis route employed. Aggressive oxidation protocols, including the classical Hummers method and its variants, typically yield C/O ratios in the range of 1.3–1.8, indicative of a high degree of basal-plane functionalization with epoxide and hydroxyl groups. In contrast, the Tour method produces graphene oxide (GO) with C/O ratios of approximately 2.1–2.6, preserving a greater proportion of the sp^2^ carbon network and resulting in more ordered laminate structures with narrower interlayer spacing distributions [53,54]. Intermediate oxidation conditions yield ratios in the 1.8–2.1 range, providing a continuously tunable synthesis parameter for membrane design. Notably, the initial C/O ratio of the as-synthesized GO determines the upper limit of subsequent reduction tunability: Tour-derived GO retains more reducible sp^2^ domains, whereas heavily oxidized Hummers GO contains a higher density of irreducible defect sites that persist even after aggressive reduction [54].

Raman spectroscopy is the primary non-destructive tool for tracking the structural evolution from GO to rGO [55]. The D band (~1350 cm^−1^), activated by sp^3^ defects and edge sites introduced during oxidation, and the G band (~1580 cm^−1^), arising from in-plane E_2_g vibrations of sp^2^ carbon, together define the I_D/I_G ratio as a quantitative measure of defect density. As-synthesized GO typically exhibits I_D/I_G values of 0.8–1.0. Upon chemical, thermal, or photochemical reduction, a counterintuitive increase in I_D/I_G to 1.0–1.4 is frequently observed, attributable to the nucleation of numerous small, newly restored sp^2^ domains whose edges contribute disproportionately to the D band relative to their interior area, a phenomenon described by the Tuinstra–Koenig relation in its inverse regime [56,57]. Progressive annealing above 800 °C or multi-step chemical reduction can ultimately decrease I_D/I_G below 0.5, approaching the defect densities of CVD-grown graphene. The partial recovery of the 2D band (~2700 cm^−1^) and the G/2D intensity ratio further report on layer stacking order and electronic conjugation restoration, both of which correlate with interlayer transport resistance in laminate membranes [58].

X-ray photoelectron spectroscopy (XPS) C1s peak deconvolution provides the chemical-state resolution that Raman spectroscopy cannot access, enabling direct quantification of individual oxygen functional group populations. The C1s envelope of GO is routinely resolved into five components: sp^2^/sp^3^ C–C at ~284.6 eV, C–O (epoxide and hydroxyl) at ~286.5–286.8 eV, C=O (carbonyl) at ~287.8–288.0 eV, O–C=O (carboxyl) at ~288.5–289.0 eV, and the π→π* shake-up satellite at ~290–291 eV [59]. It should be noted, however, that C1s deconvolution is notoriously sensitive to peak-fitting constraints, specifically the number of components included, the Gaussian/Lorentzian mixing ratio, and the choice of background subtraction method (Shirley vs. Tougaard), and that inter-laboratory comparisons of absolute component percentages should be interpreted with caution unless identical fitting protocols are applied. In as-synthesized GO, the C–O component typically accounts for 40–55% of the total C1s area, while the sp^2^ peak is suppressed to 25–35% [60]. Upon reduction, the progressive attenuation of the C–O peak and concurrent growth of the sp^2^ component and π→π* satellite confirm the selective removal of basal-plane epoxides and hydroxyls. Importantly, XPS deconvolution consistently reveals that carboxyl groups at sheet edges are substantially more resistant to reduction than basal-plane epoxides, a finding with direct implications for membrane surface charge and interfacial water structuring. The atomic C/O ratio derived from the XPS survey spectrum serves as the quantitative ground truth for synthesis-method comparisons, directly linking chemical state to transport-relevant structural parameters, such as interlayer spacing and hydration capacity [37].

Partial chemical, thermal, or photochemical reduction in graphene oxide (GO) progressively restores sp^2^ character, enhances hydrophobicity, and reduces interlayer spacing, enabling transport properties tunable across the continuum between GO and pristine graphene. The degree of reduction can be precisely monitored through the combined use of Raman I_D/I_G ratios and XPS C1s peak area ratios, providing complementary structural and chemical-state information that neither technique can supply alone. This dual-characterization approach has become the standard for reporting rGO quality in membrane studies published in high-impact venues and should be adopted as the baseline characterization protocol for GO-based membrane materials.

The lateral size and thickness of GO and rGO sheets are not fixed material constants but synthesis- and processing-dependent variables with direct consequences for laminate transport performance. Lateral sheet dimensions in membrane-relevant GO dispersions span a wide range: Hummers-derived GO produced from natural flake graphite typically yields sheets of 1–10 μm lateral size, while sonication-assisted exfoliation reduces this to 0.1–1 μm, and electrochemical exfoliation can produce sheets up to 20–50 μm with lower defect density. Tour-method GO tends toward intermediate lateral sizes (2–8 μm) with more uniform oxidation distribution. In the membrane context, lateral sheet size governs the effective tortuosity of the transport path: larger sheets require permeating molecules to travel longer lateral distances between sheet edges before advancing one interlayer step, increasing the tortuosity factor τ and consequently the τ^2^/ε correction to MD permeance predictions discussed in Section 3.4. Specifically, for a laminate membrane of thickness δ assembled from sheets of average lateral dimension L, the minimum number of interlayer channel-traversal steps scales as δ/tso (where tso is the interlayer spacing), while the additional lateral path per step is proportional to L/2 in the absence of sheet overlap. Consequently, membranes assembled from large sheets (L > 5 μm) show experimentally lower permeance for a given thickness than those assembled from small sheets (L < 0.5 μm) under otherwise identical conditions, a trend consistent with the tortuosity framework and reported by Akbari et al. [39]. for shear-aligned GO laminates across the 0.5–10 μm size range. For rGO, chemical or thermal reduction does not significantly alter lateral sheet dimensions, but it narrows the interlayer spacing from 0.8–0.98 nm (hydrated GO) to 0.34–0.6 nm and reduces the fraction of oxygenated boundary regions, shifting the transport regime balance toward the viscous/near-frictionless flow component of the three-regime model.

The degree to which GO or rGO sheets are aligned parallel to the membrane plane, and how uniformly they are dispersed throughout the laminate, critically determines whether the tortuous interlayer pathway that provides selectivity is well-defined and reproducible, or whether misaligned sheets and aggregates create non-selective through-pores. Three distinct alignment regimes are observed depending on the assembly method and GO concentration. (1) Isotropic dispersion: at low GO concentrations (<0.5 mg/mL) and without directional deposition forces, sheets adopt random orientations in the deposited film, producing a laminate with broad d-spacing distribution and high defect-pathway density. This regime is undesirable for selective membranes and is characteristic of dilute spray-deposited films without post-deposition compression. (2) Partial nematic alignment: GO dispersions above a critical concentration (~2–5 mg/mL for sheets of >1 μm lateral size) spontaneously form discotic nematic liquid crystal phases, as demonstrated by Akbari et al. [39], in which sheet normals align preferentially perpendicular to the membrane plane. Vacuum filtration, pressure-assisted self-assembly, and slot-die coating exploit this liquid-crystalline behavior to produce laminates where the majority of sheets are within ±15° of the membrane plane, as confirmed by grazing-incidence X-ray diffraction (GIXRD) and cross-sectional TEM. This alignment regime produces the narrowest d-spacing distributions and lowest non-selective defect densities reported for GO laminates. (3) Enhanced alignment via shear: doctor-blade, slot-die, and R2R coating methods apply shear during deposition, further increasing Herman’s orientation parameter S (typically S = 0.6–0.85 for shear-aligned GO vs. S = 0.3–0.5 for unsheared filtration-deposited films). Higher S correlates directly with lower salt passage and higher rejection in NF applications. For rGO, thermal or chemical reduction after assembly partially disrupts liquid-crystalline alignment due to re-aggregation driven by restored hydrophobic basal planes; cross-linking before reduction (e.g., with glutaraldehyde or boric acid) suppresses this re-aggregation and preserves alignment. The relationship between sheet dimensions, alignment state, d-spacing distribution, and transport performance therefore represents an integrated structural design parameter set, not independently adjustable variables, and should be co-reported in membrane characterization protocols alongside C/O ratio and interlayer spacing.

## 3. Mechanisms of Molecular and Ionic Transport

### 3.1. Transport Through Nanoporous Graphene

In single-layer nanoporous graphene (NPG) membranes, transport occurs exclusively through intentionally introduced pores of sub-nanometer to nanometer dimensions. The separation mechanism primarily relies on size exclusion at the atomic scale, complemented by electrostatic and chemical interactions between permeating species and pore-edge functional groups. Due to the membrane’s extremely thin thickness, the transport resistance is minimized to the theoretical minimum of the pore itself. This characteristic confers the potential for exceptionally high permeability, orders of magnitude surpassing conventional membranes [61].

The selectivity of NPG membranes is highly sensitive to the pore size distribution. Molecular dynamics (MD) simulations have demonstrated that pores of approximately 2.75 Å diameter can facilitate the passage of water molecules while rejecting hydrated ions, whereas pores of approximately 5.5 Å can distinguish between ions based on their hydrated radius. In practical terms, achieving such precise and monodisperse pore size distributions over macroscopic areas remains a significant fabrication challenge. Pore creation methods encompass electron beam irradiation, ion bombardment, UV-induced oxidative etching, and chemical etching, each with distinct trade-offs in terms of pore density, size control, and membrane area [41,42,62,63].

### 3.2. Transport Through GO Laminates

In GO laminate membranes, transport does not occur through discrete pores in individual sheets but rather through a complex, hierarchically structured network of interlayer nanochannels formed between stacked GO sheets [9]. Permeating species navigate a tortuous path comprising two structurally distinct domains: the pristine graphitic regions, presenting a low-friction hydrophobic surface that functions as an express lane for water molecules. Additionally, oxygenated regions constrain passage through steric, energetic, and electrostatic interactions [64]. The interlayer d-spacing, typically 0.7–1.0 nm under wet conditions and expanding to 1.1–1.8 nm under full immersion, depending on oxidation degree and ionic environment, defines the geometric envelope within which all transport phenomena operate [65].

The seminal work of Nair et al. (2012) [9] established that graphene oxide (GO) membranes exhibit anomalously rapid water vapor permeation while remaining essentially impermeable to all other gases, including helium. This striking selectivity is attributed to the formation of a low-friction water monolayer within the graphitic interlayer galleries. This observation immediately raised a mechanistic question that remains unresolved: is the extraordinary permeance of GO laminates a consequence of solution–diffusion through a hydrophilic matrix, viscous pressure-driven flow through nanoslit channels, or activated molecular hopping across energy barriers imposed by the oxygenated domains? These three frameworks each carry independent experimental and computational support, and critically, they are not mutually exclusive.

In the solution–diffusion model, water and solutes dissolve into the GO matrix at the upstream face and migrate down a chemical potential gradient, with flux governed by permeant solubility and diffusivity in the interlayer environment. Temperature-dependent permeation experiments yielding activation energies of 15–30 kJ mol^−1^ for water transport are broadly consistent with diffusion through a hydrophilic matrix, supporting this interpretation [43,66]. However, solution–diffusion theory cannot account for permeance values orders of magnitude above what diffusivity through an equivalent-thickness dense polymer membrane would predict [67,68], nor for the dynamic swelling and collapse of the interlayer channel in response to pressure, ionic strength, and humidity behavior that a rigid solution–diffusion framework has no mechanism to describe [9,12].

The viscous flow model instead treats GO interlayer channels as slit-shaped nanopores through which a hydraulic pressure gradient drives water according to a modified Hagen–Poiseuille relation for confined geometry. The inverse scaling of permeance with membrane thickness reported in several studies, and molecular dynamics evidence for a highly mobile, structurally ordered water phase in GO channels, are consistent with this picture [69]. Nevertheless, applying a continuum viscous flow framework to channels of 0.7–1.2 nm width where water occupies only one to two molecular layers and continuum fluid mechanics loses physical validity is fundamentally inconsistent with the assumptions underlying Hagen–Poiseuille theory. A third framework, activated hopping, attributes both permeance and selectivity to discrete molecular-scale energy barriers arising from partial dehydration, steric constriction near functional group sites, and electrostatic interactions with the GO surface. The observed K^+^ > Na^+^ > Li^+^ permeation sequence, which correlates with partial dehydration energy rather than bare or hydrated ion size and Arrhenius-type activation energies of 20–50 kJ mol^−1^ measured for multivalent ion transport, is most naturally explained within this framework [12]. The emerging consensus reconciles all three models by invoking the lateral structural heterogeneity of GO sheets: viscous or near-frictionless flow dominates within the pristine graphitic corridors, activated hopping governs passage across the boundaries of oxygenated constriction sites, and solution–diffusion provides the overarching thermodynamic driving force that couples these two parallel pathways. This three-regime picture carries direct design implications: maximizing the proportion of pristine graphitic corridors through synthesis control of the C/O ratio enhances permeance, while engineering the oxygenated domain boundaries through selective functionalization tunes selectivity, offering a rational strategy for decoupling these two historically coupled properties.

The three-regime model provides the first explicit reconciliation of three previously competing frameworks. (1) The solution–diffusion model [67] correctly predicts thermodynamic driving force and activation energies of 15–30 kJ/mol for water transport but cannot account for permeances orders of magnitude above equivalent-thickness dense polymers. (2) The viscous/Hagen–Poiseuille framework [69] explains inverse thickness–permeance scaling but loses physical validity at channel widths of 0.7–1.2 nm where continuum fluid mechanics breaks down. (3) The activated hopping framework [70] explains the K^+^ > Na^+^ > Li^+^ permeation sequence and Arrhenius energies of 20–50 kJ/mol for multivalent ions, but does not account for anomalously high water permeance. The model’s predictive consistency is supported by: Abraham et al. [12] (d-spacing tunable 0.65–0.98 nm, selectivity shifting from monovalent to divalent exclusion); Sun et al. [71] and Chen et al. [72] (rejection collapse at low pH, consistent with the Donnan exclusion component); and Zhang et al. [73] (surface-charge-controlled rejection exceeding commercial NF limits). A formal quantitative partitioning of regime contributions as a continuous function of C/O ratio has not yet been achieved experimentally and represents an identified priority requiring GO membranes with spatially resolved C/O maps combined with permeance and selectivity measurements.

The transport properties, selectivities, and mechanistic interpretations reported across the GO membrane literature vary considerably between research groups, and this variability is not fully explained by random experimental error. Several systematic sources of disagreement can be identified. Differences in GO synthesis route (Hummers, modified Hummers, Tour, electrochemical) produce different C/O ratios, oxygen functional group distributions, and lateral sheet sizes for nominally similar starting materials, which propagate directly into differences in measured d-spacing, permeance, and selectivity. Differences in membrane fabrication method (vacuum filtration, pressure-assisted assembly, slot-die coating, spray deposition) affect tortuosity and defect density independently of the intrinsic GO sheet chemistry, so that two membranes with identical C/O ratios can show substantially different transport behavior purely as a consequence of assembly method. Differences in testing conditions, including applied pressure, feed concentration, pH, ionic strength, and temperature, are often incompletely standardized or reported across studies (see also Section 6), complicating direct comparison of quantitative performance metrics. Finally, differences in characterization methodology used to report structural parameters, for example, whether d-spacing is measured in the dry or hydrated state, or whether C/O ratio is determined by XPS or elemental analysis, can produce systematic offsets between studies that are sometimes mistaken for genuine material differences. We highlight these sources of variability explicitly where relevant throughout the mechanistic and application Sections below, rather than treating reported values as directly comparable across studies by default.

The apparent sieving behavior of GO laminates, most prominently the ~4.5 Å ionic radius cut-off reported by Joshi et al., 2014 [8], must be understood within this multi-mechanism context rather than as a simple geometric sieve. Joshi et al. interpreted the sharp permeation threshold as evidence of steric exclusion by a geometrically defined interlayer channel, a conclusion subsequently contested on mechanistic, electrostatic, and generalizability grounds by multiple independent groups [12,70,71] and others, and should not be treated as universally valid. Three lines of experimental evidence challenge the purely steric interpretation. First, partial dehydration energy barriers have been shown to govern ion-specific selectivity in a manner that steric exclusion alone cannot explain: Shi et al., 2017 [70] demonstrated via MD simulation that the free energy barrier for ion passage through GO constrictions is dominated by the energetic cost of partial hydration shell removal, accounting for the observation that divalent ions of similar hydrated radius to monovalent species are rejected at disproportionately higher rates. A study by Zhang et al., 2019 [73] demonstrates that GO membrane surface charge directly controls ion rejection, with the highly charged GO surface repelling high-valence co-ions through interaction energy barriers while restraining counter-ion permeation to maintain overall charge balance, achieving rejection performance exceeding the limits of commercial nanofiltration membranes; this is the most direct experimental proof that rejection scales with ionic charge in GO membranes under pressure-driven conditions. Second, electrostatic Donnan exclusion has been demonstrated to contribute substantially to the apparent selectivity: Sun et al., 2016 [71], showed that ion rejection by GO membranes collapses sharply at low pH when carboxylate groups are protonated, and membrane surface charge is neutralized, a behavior that is irreconcilable with a steric mechanism but fully consistent with charge-controlled exclusion. Chen et al., 2017 [72], independently confirmed this by demonstrating that chemical methylation of edge carboxylate groups reduced ion rejection for species with hydrated radii well below the proposed 4.5 Å steric cut-off, directly falsifying the steric-only interpretation for that ion population. Third, the universality and reproducibility of the 4.5 Å threshold have been directly questioned: Abraham et al. (2017) [12] demonstrated that epoxy cross-linking can shift the effective cut-off continuously across a range of d-spacings from 6.4 to 9.8 Å, establishing it as a preparation-specific parameter rather than an intrinsic characteristic of GO laminates. Abraham et al., 2017 [12], further demonstrated that epoxy cross-linking can be used to deliberately tune the effective cut-off across a continuous range, confirming that the Joshi value represents one point in a tunable parameter space rather than a fundamental physical threshold.

The driving forces for transport in GO laminates encompass hydraulic pressure gradients (NF and RO applications), concentration gradients (dialysis and ion diffusion), and electrochemical potential differences (electrodialysis and osmotic energy harvesting). In all instances, the apparent permeability and selectivity are determined by the synergistic interplay of channel geometry (interlayer spacing, sheet lateral dimensions, and degree of sheet overlap), surface chemistry (hydrophobicity, charge density, and functional group distribution), and permeant properties (molecular size, charge state, hydration energy, and chemical affinity for the GO surface). The mechanistic debates reviewed above converge on a singular practical conclusion: selectivity in GO laminates is not a fixed material property but an emergent, adjustable function of synthesis conditions, environmental variables, and operating parameters. Consequently, achieving the simultaneous high permeance and high selectivity required by next-generation membrane applications necessitates the coordinated optimization of interlayer spacing, surface charge density, and functional group chemistry parameters that are independently accessible through the synthesis and functionalization strategies elucidated in Section 5.

We note that the relative contributions of steric exclusion, Donnan exclusion, and partial dehydration to ion sieving in GO laminates remain a genuinely unresolved disagreement in the field rather than a settled question; the experimental conditions under which each mechanism’s supporting evidence is strongest (e.g., low ionic strength favoring Donnan exclusion, narrow d-spacing favoring steric and dehydration effects) differ across the studies cited here, and we have refrained from presenting any single mechanism as dominant across all reported conditions.

### 3.3. Computational Modeling of Transport: A Roadmap from Classical to Quantum Methods

Computational modeling has emerged as an indispensable cornerstone of graphene-based membrane research, offering mechanistic insights into transport phenomena that remain experimentally inaccessible at the atomic scale. Given the rapid evolution of simulation methodologies, this Section serves as a forward-looking computational roadmap, surveying the current state of each modeling tier, critically evaluating their limitations in the context of GO membrane transport, and identifying the methodological transitions required to resolve the mechanistic debates outlined in Section 3.2. Readers primarily interested in fabrication and applications may proceed to Section 4.

Classical molecular dynamics employing validated force fields (SPC/E, TIP4P, OPLS-AA) remains the primary workhorse for semi-quantitative predictions of water permeance and ion rejection in GO systems [69,74]. Its principal limitation for GO membranes is systematic: SPC/E overestimates confined water diffusivity by 30–50% relative to higher-fidelity references [75,76], and the idealized channel geometry assumed in simulation, absent tortuosity, interlayer misalignment, and partial channel blockage, produces permeance predictions that consistently exceed experimental values [39,77,78]. The design implication is direct: classical MD is reliable for rank-ordering membrane compositions and identifying qualitative permeance trends, but quantitative targets for engineering design should be derived from experimentally calibrated continuum models rather than raw MD output [12,79].

Machine-learning force fields (ML-FFs) represent a promising but still early-stage methodology for GO and carbon–water systems, with documented applications to date remaining limited to a small number of research groups and systems. Across the principal GO-relevant ML-FF developments reported so far, including GO-specific Gaussian Approximation Potentials [80], committee neural network potentials for confined water, ion-transport ML-FFs for nanoporous carbon channels, and the emerging MACE architecture [81,82], a consistent picture emerges across the limited number of published studies: ML-FFs correctly reproduce the anisotropic mechanical and electrostatic landscape introduced by GO functional groups, capture preferential water clustering around epoxide sites at low C/O ratios that supports the activated hopping mechanism of Section 3.2, and reduce the free-energy barrier errors for ion passage that classical force fields systematically accumulate. The design implication is that ML-FFs hold promise for resolving mechanism debates where classical MD predictions are demonstrably biased, particularly the quantitative partitioning between viscous-flow and activated-hopping regimes across C/O ratio space; however, classical MD remains the dominant tool in practice for the foreseeable future, and ML-FF adoption is currently restricted by the computational and expertise barriers discussed below.

Training a GO-specific GAP or MACE model necessitates a reference dataset comprising 5000 to 50,000 DFT single-point calculations (requiring several thousand CPU-hours) and expertise in hyperparameter tuning, which is not commonly found within membrane science research groups. Transferability across C/O ratios is not assured without retraining. For research groups interested in implementing these methods, the following open-source resources are available: the QUIP/quippy framework (libAtoms/QUIP, GitHub, quippy-ase v0.10.3) provides the reference GAP implementation with GO-relevant example workflows; the MACE architecture (ACEsuit/mace, GitHub, v0.3.16) is fully open-source with documented MD tutorials; the NOMAD repository (https://nomad-lab.eu/) and the Materials Project (https://next-gen.materialsproject.org/) host DFT datasets for carbon-based materials suitable as starting-point training sets; and the OpenKIM project (https://openkim.org/) provides a standardized testing framework for interatomic potential validation. For most membrane research groups, the practical entry point is to utilize published GO-specific ML-FF parameters for screening and reserve force-field training for novel compositions where classical MD demonstrates demonstrable bias.

Nuclear quantum effects (NQEs), arising from the wave-like nature of light nuclei at sub-nanometer confinement, are application-dependent in their significance. For water permeance and monovalent ion rejection, path-integral simulations of bulk water demonstrate that NQEs increase water diffusivity by approximately 15–50% relative to classical MD, depending on the force field and methodology, with more accurate ab initio PIMD studies converging on the lower end of this range (~15%) [83,84,85]; whether this correction applies quantitatively to sub-nanometer GO interlayer channels remains an open question, as NQEs in confinement are geometry- and surface-chemistry-dependent in ways that bulk estimates do not capture, and no dedicated PIMD study of GO laminate permeance has yet been reported. For salt rejection, quantum corrections to ion free-energy barriers remain within the typical force-field uncertainties, confirming that classical MD is adequate for semi-quantitative predictions in this regime.

Practical implication: For the majority of GO membrane applications, water purification, nanofiltration, and ion sieving, classical MD with a well-validated force field remains sufficient, provided flux values are treated as upper bounds. For proton transport applications relevant to osmotic energy harvesting and proton exchange membranes, however, classical MD is expected to underestimate proton mobility due to the absence of tunneling contributions, with published path-integral studies of confined water wires reporting NQE-driven enhancements to proton diffusion [84]; the quantitative magnitude of this underestimation in GO interlayer channels specifically has not been directly measured, and path-integral methods or NQE-capable ML-FFs are recommended for quantitatively reliable predictions in this regime. It should be noted that proton transport through GO interlayer channels proceeds via Grotthuss-type hopping through hydrogen-bonded water networks, mechanistically distinct from quantum tunnelling through the sp^2^ electron density barrier reported for monolayer graphene, and the two should not be conflated when evaluating the adequacy of classical MD.

The distinction is not one of degree but of mechanism. For water molecule permeance, transport is governed by molecular diffusion dominated by oxygen (mass 16 amu) and collective molecular displacement. NQEs contribute a moderate ~15–25% correction to water self-diffusivity, as established by ab initio PIMD studies [83,85]; whether this applies quantitatively in sub-nanometer GO channels is unresolved because NQEs in confinement depend on confining-potential curvature in ways bulk estimates do not capture. The practical implication is that classical MD water permeance predictions may be underestimated by ~15–25% from NQE omission, partially offsetting the 3–8× overestimate from idealized geometry. For proton transport, the mechanism is qualitatively different: protons traverse GO channels via Grotthuss-type hopping through hydrogen-bonded water wires, where the rate-limiting step is sequential O–H bond breaking and forming by individual protons (mass 1 amu). Here, quantum tunnelling and zero-point energy differences (H/D isotope effect ~1.4–2.0 in proton conductance) are dominant mechanistic contributions. Rossi et al. [84] demonstrated via PIMD that NQEs enhance proton diffusion along confined water wires by factors of 2–4 relative to classical MD, an enhancement of the same order as the quantity being predicted, making classical MD mechanistically incomplete (not merely imprecise) for proton transport. This is why path-integral methods are mandatory for proton transport applications but optional for water permeance in GO laminate geometry.

**Figure 1:**
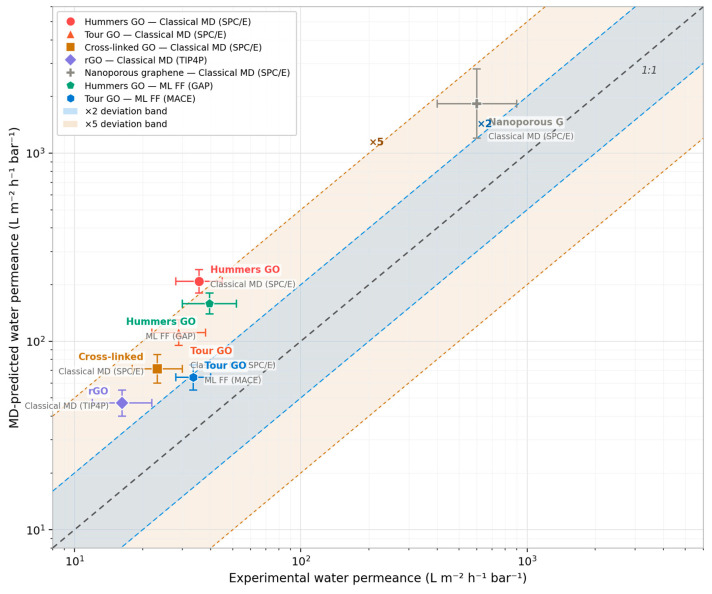
Parity plot of MD-predicted vs. experimentally measured water permeance for graphene-based membranes of varying C/O ratio and d-spacing. Data derived from Table 2. Individual data points are labelled by membrane type (Hummers GO, Tour GO, cross-linked GO, rGO, nanoporous graphene) and simulation method. Error bars represent the full range of values reported across independent studies using nominally equivalent membrane preparations. The solid diagonal denotes perfect agreement (1:1). Dashed and dotted lines denote ×2 and ×5 deviation bands, respectively. All classical MD results fall above the ×2 band, consistent with systematic SPC/E overestimation of confined water diffusivity. ML force-field predictions (GAP, MACE) fall within the ×2 band.

A critical but frequently overlooked test of any transport model is its quantitative agreement with experimental permeance data. Figure 1 presents a parity plot of MD-predicted versus experimentally measured water permeance for GO membranes of systematically varied C/O ratio and d-spacing; Table 2 summarizes the underlying dataset. Classical MD consistently overpredicts water permeance by a factor of 3–8×, attributable primarily to SPC/E diffusivity overestimation and idealized channel geometry. ML force-field predictions (GAP, MACE) narrow this gap to within ×1.5–2 of experimental values, while path-integral methods are expected to reduce the remaining discrepancy for proton-conducting channels, though the magnitude of this correction in GO laminate geometry has not yet been directly quantified. Importantly, the systematic nature of the classical MD overprediction is correctable by a geometry factor τ^2^/ε, where τ is the tortuosity of the interlayer transport path and ε is the accessible porosity fraction, both estimable from XRD lineshape analysis, bringing predicted permeances into reasonable agreement with experiment without refitting the force field itself.

The MD–experiment pairs included in Figure 1 and Table 2 were restricted to studies reporting both a classical MD-predicted permeance under a clearly stated channel geometry and a corresponding experimental permeance measurement on a structurally comparable GO laminate (consistent d-spacing range and reported C/O ratio), so that the comparison reflects matched rather than arbitrary pairings. We note, however, that the underlying experimental studies were not conducted under identical pressure, temperature, or feed conditions, and the 3–8× overprediction factor should therefore be understood as a representative range observed across the available matched comparisons rather than a precisely bounded or universally applicable correction factor. We present this range as an order-of-magnitude characterization of a systematic tendency rather than a tightly quantified result, and we encourage readers seeking to apply this correction to a specific system to consult the underlying matched studies directly for their precise testing conditions.

When considered collectively, the three modeling tiers constitute a complementary hierarchy in which each successive level rectifies specific shortcomings of the one preceding it (Table 3). For future simulation studies of GO membranes, we recommend a tiered approach: classical MD for initial screening and parameter-space exploration; ML-FF validation for key mechanistic predictions where classical force fields exhibit demonstrable bias; and path-integral corrections for any application involving proton or light-ion transport. This hierarchy reflects both the current state of the field and the computational investments now practically feasible.

Continuum models, the modified Hagen–Poiseuille equation, and the extended Nernst–Planck framework bridge the gap between atomic-scale MD insight and module-relevant scales, providing physically interpretable parameters and rational design guidance within their validity range (h > 0.6 nm; ionic strength < 0.5 M). Their parameterization and design use are detailed in Section 3.4.

The τ^2^/ε factor is not a fitted parameter but a geometrically derivable quantity estimated from wet-state XRD lineshape analysis (Scherrer equation for τ) combined with BET accessible porosity (ε). Values in Table 4 (range 4–36) span C/O ratios from approximately 1.4 (Hummers GO) to approximately 3.8 (rGO) and d-spacings from 0.65 to 0.98 nm across independent studies [12,39,89]. For rGO membranes, reduced oxygen content and improved sheet alignment typically yield τ^2^/ε at the lower end of the range (4–9). For GO composites incorporating intercalated nanoparticles (TiO_2_, MOF nodes), nanoparticle inclusion disrupts regular stacking and creates transport dead-ends; the XRD-based estimation of τ^2^/ε may underestimate the true correction, and direct back-calculation from experimental permeance data is recommended. The τ^2^/ε correction is reliable for homogeneous GO and rGO laminates but should be applied with caution and cross-checked against permeance measurements for structurally heterogeneous composite architectures.

### 3.4. Continuum Modeling: Parameterization and Design Use

Molecular dynamics simulations provide atomic-scale insight but cannot operate at module-relevant scales. Two continuum frameworks bridge this gap: the modified Hagen–Poiseuille (HP) equation for water flux and the extended Nernst–Planck (ENP) framework for ion transport. For the slit-shaped interlayer galleries of GO laminates, the HP permeance is:

(1) *Aw* = (*h*^2^/12*η*) × (*ε*/*τ^2^δ*) × 3.6 × 10^11^ [L m^−2^ h^−1^ bar^−1^]

where *h* is the open channel height (XRD d-spacing − 0.34 nm), *η* is confined-water viscosity (Pa s), *δ* is membrane thickness (m), and *τ*^2^/*ε* is the tortuosity–porosity penalty (typically 9–36 for GO laminates). The conversion factor 3.6 × 10^11^ transforms SI permeance units (m s^−1^ Pa^−1^) to engineering units (L m^−2^ h^−1^ bar^−1^), arising from the chain: × 10^3^ L/m^3^, × 3600 s/h, × 10^5^ Pa/bar. The quadratic dependence on h is the central design relationship; a 10% increase in interlayer gap raises permeance by ~21%, making swelling control a permeance design variable as much as a stability concern. The *τ*^2^/*ε* group explains why correcting idealized MD predictions, which implicitly assume τ = 1, ε = 1, by this factor brings simulated permeances into agreement with experimental values documented in Table 2.

Ion rejection is governed by the combined steric and Donnan partition coefficients at the channel entrance. The observed rejection *R*_i is:

(2) *Ri* = 1 − (*Ks*,*i* × *KD*,*i*)/[1 − (1 − *Ks*,*i KD*,*i*) *exp*(−*Pei*)]

where the steric coefficient *K*_s,i = (1 − *r*_s,i/*h*)^2^ (*r*_s,i = hydrated ionic radius of species *i*), the Donnan coefficient *K*_D,i = exp(−*z*_i*F*Δ*ψ*_D/*RT*) (Δ*ψ*_D = Donnan potential computed from fixed surface charge density *σ* and feed ionic strength), and Pe_i = *J*_v*δ*/*D*_i,eff is the ion Péclet number. The pressure-dependence of rejection, rising toward a partition-limited plateau at high Pe, is the characteristic ENP signature and the primary model validation test. The surface charge density *σ* required for *K*_D is obtained from streaming potential measurements at operating pH and ionic strength, converted via the Grahame equation:

(3) *σ* = (8*ε*_0_*εr RT c∞*)^(1/2) × *sinh*(*Fζ*/2*RT*)

where *c*_∞ is the bulk electrolyte concentration (mol m^−3^) and *ζ* is the measured zeta potential.

The three experimentally critical inputs to Equations (1) and (2) are summarized in Table 4, together with the recommended measurement protocol and typical values reported in the GO laminate literature. Two measurement details warrant particular emphasis. First, *h* must be determined from wet-state XRD at operating ionic strength; dry-state measurements underestimate *h* by 0.3–0.6 nm and cause Equation (1) to underpredict permeance by up to an order of magnitude. Second, *σ* must be measured at the operating pH, since GO carboxylate groups (pK*a* ≈ 4–5) lose their charge below pH 5, and the *σ*–*ζ* relationship in Equation (3) is strongly non-linear at high surface charge densities.

The ENP framework is valid for *h* > 0.6 nm and ionic strengths below ~0.5 M; below these thresholds, molecular layering and ion–ion interactions invalidate continuum assumptions and the atomistic methods of Section 3.3 must be substituted. Within its validity range, the recommended design workflow is: measure *h*, *τ*^2^/*ε*, and *σ* experimentally → use MD for confined viscosity and ion hindrance factors → solve Equations (1) and (2) across the target d-spacing range to map the permeance–selectivity frontier before fabrication, replacing trial-and-error iteration with targeted, predictive design.

Taken together, the multi-scale framework assembled in Section 3.2, Section 3.3 and Section 3.4, from atomistic force-field hierarchies through continuum transport parameterization, enables GO membrane permeance and selectivity to be prescribed from measurable structural inputs rather than discovered by trial-and-error fabrication. As the field moves from proof-of-concept demonstrations toward rational, application-specific membrane design, this quantitative translation between molecular structure and engineering performance is not a peripheral methodological detail but a prerequisite for accelerating the development cycle and for making credible, experimentally falsifiable predictions that distinguish genuine mechanistic understanding from post hoc data rationalization.

## 4. Membrane Fabrication Strategies

### 4.1. Free-Standing GO Laminates

The most straightforward approach to GO membrane fabrication involves vacuum filtration of aqueous GO dispersions through a porous support (typically anodized alumina, polycarbonate track-etch, or nylon membranes), resulting in a laminated GO film of controllable thickness. Spray coating, spin coating, and layer-by-layer (LbL) assembly offer complementary fabrication routes with different trade-offs in thickness control, scalability, and defect density [89]. LbL assembly, in particular, allows precise nanoscale thickness control and the incorporation of oppositely charged polycations between GO layers, providing additional functionalization and interlayer spacing control. Pressure-assisted self-assembly techniques can achieve highly aligned laminar structures with improved mechanical properties, owing to the near-parallel sheet orientation imposed by the directional flow during filtration [43,90,91].

### 4.2. Nanoporous Graphene Membranes

Single-layer or few-layer nanoporous graphene membranes are fabricated by CVD growth, transfer to a support with microscale apertures, and subsequent controlled nanoporation. Pore-creation methods, UV-induced oxidative etching, ion bombardment followed by chemical etching, and focused ion beam milling [92], each offer distinct trade-offs in size control, pore density, and throughput; no single method yet achieves monodisperse pore arrays beyond sub-cm^2^ areas (see Table 1 for a synthesis-route comparison). A persistent challenge is the presence of transfer-induced defects (grain boundaries, tears, contaminants) that create non-selective bypass pathways. Decoupling defect sealing from deliberate nanopore introduction, through nylon or hafnium oxide ALD passivation before poration, has achieved sub-nanometer MWCO membranes in laboratory settings [41].

### 4.3. Composite and Hybrid Membrane Architectures

Graphene oxide (GO) has been incorporated as a selective layer atop conventional polymer supports (polysulfone, polyethersulfone, polyacrylonitrile) via direct coating or covalent grafting, producing GO-TFC membranes with enhanced mechanical integrity and processability.

Mass transport through GO-TFC architectures is governed by a resistance-in-series framework in which the total hydraulic resistance comprises contributions from the selective GO layer, the intermediate gutter layer (where present), and the porous polymeric support. In commercial polyamide TFC membranes, the support layer resistance is typically negligible relative to the dense selective layer; however, for ultrathin GO selective layers, which can be deposited at thicknesses of 20–200 nm, far thinner than their polyamide counterparts, the intrinsic resistance of the porous support becomes a non-negligible fraction of the total membrane resistance and can significantly limit the experimentally realized permeance below the intrinsic [67,93]. Quantitatively, support layer resistance has been shown to account for 30–60% of total hydraulic resistance in GO-TFC configurations where the selective layer thickness falls below 50 nm, a finding with direct implications for support material selection and pore architecture optimization [94]. A second transport consideration specific to TFC architectures is concentration polarization within the support layer pores, a phenomenon that reduces the effective driving force across the selective GO layer and becomes particularly severe under high-flux conditions. Internal concentration polarization in FO configurations has been shown to reduce the effective osmotic driving force by 40–70% relative to bulk conditions, depending on membrane structural parameter and draw solution identity [95,96], representing a dominant performance-limiting mechanism that is absent in freestanding GO membrane configurations and must be explicitly accounted for in module-level modeling. Finally, the interfacial adhesion between the GO selective layer and the polymeric support critically determines both transport and stability: covalent grafting strategies, including amide bond formation between GO carboxyl groups and amine-functionalized supports, have been shown to reduce selective layer delamination under hydraulic pressure cycling while simultaneously improving GO layer uniformity and reducing defect-mediated transport bypass pathways [97]. Collectively, these transport considerations establish that the performance of GO-TFC membranes cannot be predicted from GO layer properties alone, and that support architecture, interfacial chemistry, and operating mode must be co-optimized to realize the intrinsic transport advantages of the GO selective layer at the module scale.

Mixed-matrix membranes (MMMs) incorporate graphene or GO as a dispersed filler within a continuous polymer matrix, simultaneously enhancing permeability (preferential transport pathways), selectivity (narrower channel-size distribution), and mechanical properties (reinforcing effect of high-aspect-ratio 2D fillers).

## 5. Functionalization and Structural Engineering Strategies

### 5.1. Interlayer Spacing Control

GO laminates undergo three physically distinct swelling modes, each requiring a separate mitigation strategy. Crystallographic swelling, d-spacing expansion from ~0.8 nm (dry) to ~1.4 nm (hydrated) driven by water intercalation, is suppressed by partial reduction [9,12]. Osmotic swelling, driven by intercalation of hydrated ions into the gallery, is best addressed by covalent cross-linking. XRD studies of GO membranes filtered with mono- and divalent electrolytes show that Ca^2+^ and Mg^2+^ expand d-spacing relative to the dry-state baseline through intercalation of their hydration shells, while monovalent Na paradoxically causes the most severe gallery expansion in uncross-linked membranes^+^ via diffuse double-layer effects; divalent ions partially bridge GO sheets and can moderate expansion at equivalent ionic strength [98]. For cross-linked membranes, however, the larger hydration shells of Ca^2+^ and Mg^2+^ present a distinct challenge, as cross-linking strategies sufficient to suppress monovalent-ion-driven swelling may inadequately restrain the hydration water of intercalated divalent species [98]. Mechanical delamination, adhesion failure at the laminate–support interface under hydraulic pressure, requires physical confinement within a rigid polymer matrix or between constraining porous substrates [43,99]. Mismatching strategy to mechanism accelerates membrane failure [100].

Precise d-spacing control is the most critical design parameter in GO laminates, governing size selectivity directly. Reduction using hydroiodic acid, ascorbic acid, or hydrazine progressively removes oxygen functional groups and reduces d-spacing while preserving sufficient hydrophilicity for water transport. Covalent cross-linking through epoxide chemistry offers finer control: Abraham et al. (2017) [12] showed that increasing epoxy loading contracts d-spacing from ~0.98 nm to ~0.65 nm, sufficient to transition the sieving cut-off from monovalent permeation to near-complete divalent exclusion, establishing d-spacing as a prescribable design parameter rather than a fixed material property. Glutaraldehyde and boric acid extend this strategy across different d-spacing ranges and pH sensitivity profiles.

### 5.2. Chemical Functionalization of Graphene and GO

Chemical functionalization of GO nanochannels enables targeted modulation of surface charge, hydrophobicity, and binding affinity. Covalent routes, amidation, epoxide ring opening, and esterification yield the highest functionalization densities: amine-grafted GO membranes show significantly enhanced heavy-metal cation rejection via electrostatic attraction, while zwitterionic modification imparts antifouling resistance essential for sustained operation in complex feeds [101] (see Section 6.5). Non-covalent functionalization via π–π stacking or hydrogen bonding preserves the graphene basal plane and is preferred when electronic conductivity must be retained for electrically enhanced separations. Intercalated MOF nanoparticles introduce ordered microporous channels with precise size cutoffs; cationic polyelectrolytes invert membrane surface charge from negative to positive, enabling switchable ionic selectivity without covalent lattice disruption [102].

### 5.3. Defect Engineering in Graphene

Top-down pore-creation strategies: electron beam sculpting, focused ion beam (FIB) milling (>10 nm resolution), and block copolymer etch masks (sub-10 nm arrays), each trade spatial precision for areal coverage; none yet delivers monodisperse pore distributions at the membrane scale [63] (see Section 4.2 and Table 1). Bottom-up alternatives graphdiyne (intrinsic triangular pores of ~5.4 Å) and synthetic porous graphene analogs offer atomically uniform pore dimensions by design, circumventing the statistical variability of top-down etching, but scalable membrane integration remains at early synthesis readiness [103].

### 5.4. Stimulus-Responsive Membranes

An emerging frontier is the design of GO membranes whose transport properties can be dynamically modulated by external stimuli, enabling “smart” separation systems. pH-responsive membranes exploit the ionization of carboxylate groups (pKa ~4–5) at the sheet edges: at high pH, deprotonation increases negative surface charge and electrostatic repulsion, tightening the effective channel and enhancing cation rejection, while at low pH, protonation reduces charge and allows greater ionic passage. Temperature-responsive behavior has been achieved by grafting poly(N-isopropylacrylamide) (PNIPAM) to GO surfaces, creating membranes that switch between open and closed states at the polymer’s lower critical solution temperature (~32 °C).

Electrically gated graphene membranes represent perhaps the most sophisticated stimulus-responsive concept, leveraging the conductivity of rGO or graphene to apply electrostatic potentials that modulate ion transport in real time. Such electrokinetically driven membranes have demonstrated ionic diode behavior, ionic current rectification, and voltage-gated ion selectivity, drawing direct parallels to biological ion channels and opening prospects for bio-inspired ionic circuits.

## 6. Applications

The performance comparisons in Section 6.1, Section 6.2, Section 6.3 and Section 6.4 draw on studies conducted under heterogeneous testing conditions (pressure range 1–20 bar, temperature predominantly 20–25 °C but not uniformly reported, feed concentration from deionized water to 0.5 M NaCl, membrane thickness inconsistently reported). Full standardization across literature sources is not feasible within the scope of a narrative review. These comparisons should therefore be understood as indicative of performance potential rather than definitive head-to-head equivalence. Where possible, key benchmark studies are identified by their specific operating conditions in each subsection. The standardized 500-h hydraulic stability protocol proposed in Section 7.3 is intended to begin addressing this inter-study comparability gap for future work. Readers drawing quantitative design conclusions from the benchmarks in this Section are advised to consult the original sources for full experimental details before application.

### 6.1. Water Desalination and Purification

Water desalination presents the most prominent potential application of graphene-based membranes, driven by their exceptional water permeability and high rejection of hydrated ions. Molecular dynamics simulations have consistently predicted that nanoporous graphene membranes with pore diameters of 5–10 Å can achieve salt rejection exceeding 99% while simultaneously exhibiting water permeabilities 2–3 orders of magnitude higher than commercial reverse osmosis (RO) membranes, which operate at 1–10 L m^−2^ h^−1^ bar^−1^ [42,68]. Although experimental nanoporous graphene membranes have not yet matched these idealized predictions, primarily due to defects and pore size distribution, graphene oxide (GO) laminates have demonstrated remarkable performance in nanofiltration (NF) and loose RO regimes.

GO membranes have demonstrated water permeances of 10–100 L m^−2^ h^−1^ bar^−1^ with molecular weight cutoffs of approximately 300–1000 Da [97,104], positioning them well for the removal of micropollutants, pharmaceuticals, and dyes from water at substantially lower energy consumption compared to RO. For context, commercial NF membranes (e.g., NF270, NF90) typically deliver permeances of 5–15 L m^−2^ h^−1^ bar^−1^ with molecular weight cutoffs of 150–300 Da [105], indicating that GO membranes can match or exceed commercial permeance while operating at comparable or wider size-exclusion ranges. Long-term stability remains a critical benchmark: Hung et al. (2014) [86] demonstrated stable GO membrane performance over 240 h of continuous NF operation at 4 bar with less than 8% flux decline. These results establish a preliminary stability record. However, it must be stated explicitly that the best available GO membrane stability data, 240 h at 4 bar for a cross-linked membrane, and 30 days at ambient pressure only, fall one to two orders of magnitude short of the qualification threshold applied to commercial NF membranes, which routinely undergo > 5000 h of continuous operation under pressure before module certification [106]. The gap is not merely incremental: it represents the difference between a laboratory proof of concept and a commercially deployable technology. Until GO membranes are demonstrated at ≥1000 h under sustained hydraulic pressure with stable rejection across representative feed chemistries, claims of NF-competitive performance should be understood as performance potential rather than demonstrated equivalence.

### 6.2. Organic Solvent Nanofiltration

Organic solvent nanofiltration (OSN) is a novel separation technology with applications in pharmaceutical manufacturing, fine chemical synthesis, and edible oil processing. Conventional polymer NF membranes exhibit swelling, plasticization, and degradation in various organic solvents. Graphene-based membranes, characterized by their chemical resilience and solvent resistance, provide significant advantages in this domain. Graphene oxide (GO) membranes have been evaluated in methanol, ethanol, dimethylformamide (DMF), and acetone, demonstrating stable permeance and molecular weight cutoff in solvents where polymeric membranes fail. Reduction in GO to reduced graphene oxide (rGO), which increases hydrophobicity, further enhances compatibility with non-polar solvents and improves organic solvent permeance [43,107].

### 6.3. Gas Separation

Single-layer graphene is intrinsically impermeable to all gases; however, the introduction of Å-scale nanopores enables molecular sieving based on kinetic diameter. Molecular simulations indicate that pores of approximately 2.5–3.5 Å can selectively permeate H_2_ (kinetic diameter 2.89 Å) over CO_2_ (3.30 Å), while pores of ~3.0–4.5 Å can discriminate CO_2_ from N_2_ (3.64 Å) [108,109]. Quantitatively, predicted and early experimental studies report H_2_/CO_2_ selectivities on the order of 10^2^–10^3^ and CO_2_/N_2_ selectivities in the range of 30–200, depending on pore size and functionalization [92,110]. These values exceed the Robeson upper bound for polymeric membranes, where typical selectivities are ~10–20 for H_2_/CO_2_ and ~20–50 for CO_2_/N_2_ at comparable permeabilities [111], while graphene membranes are additionally predicted to achieve permeances exceeding 10^4^ GPU [112]. Experimental validation of extreme selectivity at the microscale has been demonstrated by Koenig et al. (2012) [110], who reported H_2_/CH_4_ selectivity greater than 10^5^ using UV-induced defects in mechanically exfoliated graphene. However, it is critical to note that this measurement was performed at the single micron-scale bubble level, representing highly localized transport through a limited number of pores and not a continuous macroscopic membrane. At the macroscale, the best available experimental result for continuous-film nanoporous graphene is that of Celebi et al. (2014) [92], who fabricated centimeter-scale doubly-clamped graphene membranes by focused ion beam drilling and measured H_2_/CO_2_ selectivities of 3–25 with H_2_ permeances of 10^4^–10^6^ GPU, substantially lower selectivity than the Koenig microscale result, directly reflecting the difficulty of achieving uniform pore size distribution at scale. Consequently, challenges in scaling, including precise control of pore size distribution, defect density, and mechanical integrity over large areas, remain unresolved. In contrast, graphene oxide (GO) laminates have been more readily implemented for gas separations, particularly for CO_2_ capture, owing to solution–diffusion transport through their polar, oxygen-functionalized interlayer galleries, which preferentially enhance CO_2_ sorption and diffusion. Experimentally, GO membranes exhibit CO_2_/N_2_ selectivities of approximately 20–40 with CO_2_ permeances on the order of 10^2^–10^3^ GPU [113,114], comparable to commercial polymer membranes. While these values approach rather than substantially surpass the Robeson bound, GO membranes offer superior thermal and chemical stability, maintaining performance under elevated temperatures relevant to industrial flue gas treatment.

### 6.4. Ion Separation and Energy Harvesting

Selective ion transport through graphene-based membranes is gaining significant attention for various applications, including lithium-ion recovery from brines (critical for battery manufacturing), monovalent/divalent ion separations for industrial process water, and osmotic energy harvesting (“blue energy”) from salinity gradients. Graphene oxide (GO) membranes with controlled d-spacing and surface charge have demonstrated Li^+^/Mg^2+^ selectivity of approximately 10 based on charge density differences [115], and Li^+^/Na^+^ selectivity of approximately 5 based on subtle differences in hydration structure [116]. The latter separation presents a particularly challenging task due to the similarity of the two ions.

Osmotic energy harvesting, which utilizes the free energy of mixing between river water and seawater, necessitates membranes with both high ionic conductance and high ion selectivity (cation over anion, or vice versa). GO-based ionic membranes with surface charge densities exceeding commercial Nafion have been demonstrated, achieving power densities of approximately 1–10 W m^−2^ at simulated river/sea salinity gradients [100]. For direct comparison, state-of-the-art commercial pressure-retarded osmosis (PRO) systems using polymeric hollow-fiber membranes typically deliver power densities of 1–5 W m^−2^ under equivalent salinity gradient conditions, establishing that the best-performing GO membranes already match or marginally exceed this commercial benchmark [117,118,119]. However, it must be noted that the highest reported GO power densities (approaching 10 W m^−2^) have been achieved under idealized laboratory conditions, small membrane areas, controlled feed chemistry, and optimized salinity gradients, and that demonstration under continuous operation at module scale, where concentration polarization and membrane fouling substantially reduce effective power output, remains an open challenge for the field.

### 6.5. GO Membranes in Context: Comparison with Other Emerging Two-Dimensional Membrane Materials

Graphene oxide is one of several two-dimensional and porous material classes under active investigation for next-generation membrane applications, and a brief comparative perspective helps to situate GO’s advantages and limitations more clearly. MXenes (e.g., Ti_3_C_2_T_x_ and related compositions) offer higher intrinsic electrical conductivity and chemically tunable surface terminations (–O, –OH, –F) that can be exploited for electrically modulated or charge-selective separations; however, MXene membranes face interlayer-spacing control challenges broadly analogous to those of GO, and many compositions exhibit limited oxidative and hydrolytic stability in aqueous environments, which currently constrains long-term operational use. Covalent organic frameworks (COFs) provide atomically precise, designer pore architectures with excellent size-selectivity and well-defined pore size distributions, in contrast to the statistically distributed interlayer channels characteristic of GO laminates; however, COF membrane fabrication at GO-comparable large areas remains considerably less mature, and synthesis typically requires more demanding reaction conditions. Metal–organic framework (MOF) membranes offer highly tunable pore chemistry through metal-node and linker selection and have achieved notable gas-separation selectivities in laboratory studies, but typically require more complex, often solvothermal synthesis routes and exhibit narrower chemical and hydrolytic stability windows than GO laminates, particularly for water-based applications. Graphitic carbon nitride (g-C_3_N_4_)-based composite membranes, frequently paired with GO in heterojunction architectures as discussed elsewhere in this review, contribute photocatalytic functionality that GO alone does not provide, enabling combined separation–degradation applications, but standalone g-C_3_N_4_ membrane transport characterization is considerably less developed than for GO. Taken together, GO membranes are not uniquely positioned among two-dimensional materials in an absolute sense, but occupy a particular niche defined by scalable, solution-processable fabrication and broadly tunable interlayer chemistry via oxidation state and functionalization; other material classes may be preferable where atomic-precision pore architecture (COFs, MOFs) or multifunctionality (g-C_3_N_4_ composites) is the primary design driver for a given application.

### 6.6. Membrane Fouling in Water Treatment Applications

Membrane fouling, the accumulation of rejected species at or within the membrane surface, leading to flux decline and selectivity deterioration, is arguably the primary operational failure mode for GO membranes deployed in water treatment, yet it has received disproportionately limited treatment in the graphene membrane literature relative to its practical significance [120]. GO membranes are susceptible to three mechanistically distinct fouling categories, each of which interacts differently with GO surface chemistry: colloidal fouling, organic fouling, and biological fouling (biofouling). Understanding these mechanisms and their GO-specific expressions is essential for designing membranes capable of sustained performance under real-world feed conditions [121,122].

Colloidal fouling occurs when inorganic particles, clay minerals, and silica colloids deposit onto the membrane surface, forming a cake layer that increases hydraulic resistance. Graphene oxide (GO) membranes exhibit a nuanced response to colloidal foulants. The intrinsic negative surface charge of GO at neutral-to-alkaline pH (arising from deprotonated carboxyl groups, pKa ~4–5) provides electrostatic repulsion against negatively charged colloids, conferring inherent colloidal fouling resistance compared to charge-neutral polymeric NF membranes [123]. However, this electrostatic protection is substantially diminished in high-ionic-strength feeds, where electrostatic double-layer compression reduces the Debye screening length and allows colloids to approach the surface within the range of van der Waals attraction. Flux decline due to colloidal cake formation on GO membranes has been reported to reach 20–40% over 10-h filtration experiments under conditions representative of secondary wastewater effluent, with near-complete flux recovery upon hydraulic backwashing, indicating reversible cake-layer fouling rather than irreversible pore blocking as the dominant colloidal fouling mechanism [89].

Organic fouling, driven primarily by natural organic matter (NOM), encompassing humic acids, fulvic acids, and polysaccharides, presents a more challenging and less reversible fouling scenario for GO membranes than colloidal fouling. Humic acid, the most extensively studied NOM foulant, adsorbs onto GO surfaces through a combination of hydrophobic interactions with the graphitic sp^2^ domains and hydrogen bonding with GO hydroxyl and epoxide groups, forming a tenacious fouling layer that persists after hydraulic cleaning [123,124]. The rate and extent of humic acid fouling is strongly dependent on solution chemistry: divalent cations, particularly Ca^2+^, act as bridging agents between humic acid carboxyl groups and GO surface functional groups, accelerating cake formation and substantially reducing the efficacy of hydraulic cleaning [125]. Compared to commercial polyamide RO and NF membranes, GO membranes exhibit comparable or slightly superior resistance to humic acid fouling under low-divalent-cation conditions, attributable to their higher surface hydrophilicity; however, under hard water conditions representative of many surface water sources, the Ca^2+^-bridging mechanism negates this advantage.

Biofouling, the colonization of the membrane surface by microorganisms and the subsequent formation of a structured biofilm, is widely regarded as the most difficult fouling type to control in water treatment membranes. Pristine GO exhibits intrinsic antimicrobial activity through oxidative stress induced by reactive oxygen species (ROS) generated at the GO surface, membrane disruption via direct physical contact with GO nanosheet edges, and inhibition of bacterial cell division [126,127]. Quantitatively, GO membranes have demonstrated 60–90% reductions in viable E. coli cell counts relative to cellulose acetate controls under equivalent exposure conditions [126]. However, this intrinsic bactericidal activity does not translate straightforwardly into biofouling resistance at the module scale: established biofilms are substantially more tolerant of antimicrobial surfaces than planktonic cells, and the extracellular polymeric substance (EPS) matrix secreted by biofilm communities provides physical shielding that attenuates direct GO-cell contact. Furthermore, reduction in GO surface functional groups, whether intentional or incidental under prolonged operation, diminishes the ROS-generating capacity and thus the antimicrobial efficacy of the membrane [124].

Antifouling surface engineering strategies for GO membranes fall into three principal categories. First, zwitterionic functionalization, briefly mentioned in Section 5.2, grafts charge-balanced moieties, such as carboxybetaine or sulfobetaine, onto the GO surface, creating a tightly bound hydration layer that sterically excludes foulant approach. Zwitterionic GO membranes have demonstrated 85–95% reduction in protein (bovine serum albumin) fouling relative to unmodified GO controls, with flux recovery ratios exceeding 95% after hydraulic cleaning, substantially superior to the 60–75% recovery typical of commercial polyamide NF membranes [128]. Second, silver nanoparticle (AgNP) incorporation into the GO matrix provides sustained antimicrobial activity through Ag^+^ ion release, with biofouling reductions of 70–95% reported against mixed microbial communities [124]; however, AgNP incorporation raises concerns about long-term Ag^+^ leaching into permeate streams and progressive loss of antimicrobial efficacy as the Ag reservoir depletes. Third, photocatalytic GO composites incorporating TiO_2_ enable UV-activated ROS generation for periodic membrane disinfection, offering a regenerable antifouling strategy that does not deplete with time [129]. Among these approaches, zwitterionic functionalization currently offers the most favorable combination of fouling resistance, cleaning efficacy, and permeance retention for GO membranes targeting water purification applications.

## 7. Challenges and Future Outlook

While the preceding Sections of this review devote substantial attention to fundamental transport mechanisms and computational modeling, we wish to emphasize here that translational and manufacturing challenges, rather than incomplete mechanistic understanding alone, are likely to represent the dominant barrier to industrial adoption of GO membranes in the near term. The subsections that follow accordingly address mechanistic understanding (Section 7.1), scalable fabrication trade-offs (Section 7.2), hydraulic stability benchmarking (Section 7.3), economic feasibility (Section 7.4), technology readiness assessment (Section 7.5), and environmental sustainability (Section 7.6), to provide a more balanced treatment between fundamental and practical considerations than is sometimes found in the literature.

### 7.1. Mechanistic Understanding

Despite significant advancements, the atomistic mechanisms underlying transport selectivity in graphene oxide (GO) membranes remain incompletely elucidated. The intricate relationship between GO chemical heterogeneity (functional group distribution, oxidation pattern), interlayer structure, and transport properties is multifaceted and not yet fully captured by existing theoretical models. Advanced characterization techniques, encompassing synchrotron X-ray scattering, solid-state NMR [130], cryo-transmission electron microscopy (cryo-TEM) [49], and X-ray photoelectron spectroscopy in conjunction with high-fidelity simulations employing machine learning force fields, are facilitating increasingly precise structural-property correlations [131] (see Section 3.3 for a detailed review of documented ML-FF studies on GO systems). Further progress necessitates the standardization of GO synthesis and membrane fabrication protocols to facilitate meaningful comparisons across research groups [106].

### 7.2. Scalable and Defect-Free Fabrication

Perhaps the most critical challenge facing graphene-based membranes is the translation of their exceptional laboratory-scale performance to large-area, defect-controlled films compatible with industrial membrane module formats. In particular, chemical vapor deposition (CVD)-grown graphene, while scalable in principle, inevitably contains grain boundaries, wrinkles, and transfer-induced defects that introduce non-selective transport pathways and significantly compromise gas selectivity. Even low defect densities can dominate transport behavior due to the atomic thickness of graphene. Graphene oxide (GO) laminates, although more readily scalable, present a different set of challenges, including intrinsic heterogeneity in flake size, degree of oxidation, and stacking order, all of which lead to poorly defined and spatially variable transport pathways. As a result, achieving uniform and reproducible separation performance remains difficult. Addressing these limitations requires the development of continuous, large-area fabrication strategies, such as roll-to-roll (R2R) processing, that can preserve precise nanoscale structural control while ensuring mechanical robustness and defect minimization. Notably, R2R manufacturing of graphene has been demonstrated through the production of 30-inch-scale monolayer graphene films via CVD [38], as well as high-speed continuous graphene growth on moving substrates using R2R CVD reactors [132]. In addition, R2R-compatible transfer and membrane fabrication strategies have achieved >99% graphene coverage on porous supports, highlighting their potential for scalable membrane production [133]. For GO membranes, scalable approaches, such as slot-die coating integrated with R2R processing, have enabled fabrication of large-area (~90 × 30 cm) membranes with controlled thickness and uniformity [134]. Despite these advances, maintaining defect-free, high-selectivity performance at industrially relevant scales remains an unresolved challenge, requiring close integration of materials synthesis, membrane engineering, and process design.

Among scalable fabrication approaches for nanoporous graphene and graphene oxide (GO) membranes, roll-to-roll (R2R) chemical vapor deposition (CVD) has been demonstrated at roll widths up to 30 inches with greater than 99% coverage on porous supports. However, transfer-induced defect densities (10^10^–10^12^ cm^−2^ for PMMA-assisted wet transfer, reduced to 10^8^–10^9^ cm^−2^ via electrochemical delamination) and high capital costs currently limit its application to pilot-scale operations, making it most suitable for applications prioritizing coverage over single-crystal perfection. In contrast, slot-die coating/R2R processing of GO laminates has achieved larger areas (~90 × 30 cm) with controlled thickness and uniformity at substantially lower cost. Individual-sheet defects are statistically mitigated through multi-sheet stacking, leaving d-spacing uniformity at scale as the primary remaining challenge. This positions it as the most immediately scalable route for water nanofiltration and ion sieving. Other methods remain more specialized: pressure-assisted vacuum filtration yields highly aligned laminates but is inherently batch-scale and best regarded as a research tool. Spray assembly shows promise for non-planar coating but lacks membrane-grade uniformity data. Layer-by-layer assembly offers precise nanoscale control at the cost of very low throughput, restricting it to specialty charge-architecture applications. Overall, slot-die/R2R GO coating offers the best near-term cost–throughput–quality balance, while R2R CVD remains the only route to single-layer nanoporous graphene, albeit at considerably higher cost.

### 7.3. Long-Term Stability

Hydraulic stability, the ability of the GO membrane to maintain flux and rejection under sustained transmembrane pressure, is the most extensively reported stability metric, though the literature remains highly heterogeneous in methodology and reporting standards. The best-documented hydraulic stability result for a cross-linked GO-TFC membrane is that of Hung et al. (2014) [86], who demonstrated less than 8% flux decline over 240 h of continuous NF operation at 4 bar with stable NaCl rejection, attributing performance retention to glutaraldehyde cross-linking that suppressed interlayer swelling. Joshi et al. (2014) [8] reported stable ionic sieving over 30-day immersion at ambient pressure, confirming structural integrity in the absence of hydraulic stress but providing no data under applied pressure. At higher pressures relevant to loose RO operation (10–20 bar), hydraulic stability data are conspicuously sparse: Tsou et al. (2015) [43] reported progressive flux decline of approximately 35% over 72 h at 15 bar for an unmodified GO laminate, with post-mortem XRD confirming partial delamination at the GO–support interface as the dominant failure mode. Cross-linking with polyethylenimine (PEI) reduced this decline to below 12% under equivalent conditions, establishing covalent interfacial bonding as the critical stabilizing strategy for high-pressure hydraulic applications. Collectively, the available data support hydraulic stability over hundreds of hours only for cross-linked GO membranes operated within the NF pressure regime (1–10 bar); stability at RO-relevant pressures over industrially meaningful timescales (>1000 h) has not been demonstrated.

Chemical stability, resistance to degradation under the cleaning agents and feed chemistries encountered in real water treatment operations, represents a distinct and more severe challenge than hydraulic stability, and has received substantially less systematic investigation. Commercial membrane cleaning protocols typically employ alternating acid (pH 2–3) and alkaline (pH 11–12) cleans, as well as oxidative cleans using sodium hypochlorite (NaOCl) at 200–500 ppm. GO membranes are fundamentally vulnerable to alkaline conditions: hydroxide ions catalyze the hydrolysis of ester and ether linkages within the GO functional group network, progressively increasing interlayer d-spacing and reducing size-exclusion selectivity [135]. Quantitatively, Chong et al. (2019) [135] demonstrated that unmodified GO membranes exposed to a pH 11 solution for 24 h exhibited a 60% increase in water permeance and a 25% reduction in dye rejection, corresponding to d-spacing expansion from 0.82 nm to 1.14 nm, as confirmed by XRD. Oxidative NaOCl cleaning poses the additional risk of further oxidizing GO functional groups and disrupting the laminate structure; systematic data on GO tolerance to chlorine-based cleaning remain limited. Chemical stability is substantially improved by covalent cross-linking: PEI- and diamine-cross-linked GO membranes retained greater than 90% of initial rejection after five consecutive acid–base cleaning cycles, where uncross-linked controls showed greater than 40% rejection loss [94,135]. Nevertheless, the chemical stability of cross-linked GO membranes under the full range of industrial cleaning protocols, including enzymatic and surfactant-based cleans, has not been systematically characterised, representing a critical data gap for commercial translation.

The absence of standardized testing conditions across GO membrane stability studies makes direct cross-group comparison largely impossible. Table 5 assembles the available hydraulic stability data from the primary literature alongside a proposed minimum benchmark protocol (final row, shaded blue). The heterogeneity of operating pressure (1–10 bar), test duration (24–240 h), and feed chemistry immediately reveals why a consensus on GO membrane durability has not emerged: no two studies share identical conditions, and the longest test (240 h, [64]) remains far short of the ≥500 h benchmark routinely applied to commercial NF/RO membranes during module qualification. Flux decline values are color-coded: **green ≤ 10%** (stable); **amber 10–25%** (moderate); **red > 25%** (severe). The adoption of the proposed standardized protocol, 0.2 M NaCl feed, 10 bar, 500 h, 25 °C, pH 6–8, as a community minimum would enable the kind of cross-laboratory comparison currently absent from the field.

The 500-h duration serves as a pragmatic minimum for cross-laboratory comparability, not a claim of sufficiency for capturing all long-term failure modes. It represents approximately twice the best current GO membrane stability record (240 h [86]) and is achievable within a single continuous laboratory test (~3 weeks). It is explicitly not a commercial certification standard: commercial NF/RO membranes undergo ≥ 5000 h of continuous pressure operation before module qualification, and the 500-h threshold falls far short of this. Regarding specific failure modes: delamination at the GO–support interface begins within 72 h at >10 bar for unmodified laminates [43]; cross-linking suppresses this within the 240-h record. Chemical reduction in GO functional groups under sustained hydraulic stress, including hydroxide-catalyzed hydrolysis of ether and ester linkages, has not been systematically characterized at any duration in the pressure-driven NF literature, and 500 h may be insufficient to capture slow chemical degradation pathways. The proposed protocol should be understood as a minimum cross-laboratory comparability standard, with the expectation that the community will extend it toward the ≥1000-h threshold needed to make credible claims of NF-competitive performance.

A significant and underappreciated gap in the GO membrane stability literature is the complete absence of standardized accelerated aging protocols analogous to those employed for commercial polymeric membranes. For polyamide RO and NF membranes, accelerated aging is routinely conducted using elevated-temperature immersion (70–80 °C), combined pressure–temperature cycling, or accelerated chlorine exposure calibrated to equivalent service-life using established degradation models [138]. No equivalent standardized protocol exists for GO membranes, and the stability data published to date are not directly comparable across research groups due to variability in test duration (24 h to 30 days), applied pressure (0–15 bar), feed chemistry, and failure metric. The absence of standardization presents a significant impediment to technology readiness assessment. Without a universally accepted accelerated aging methodology, it becomes challenging to conduct meaningful stability comparisons between GO membrane variants and translate laboratory stability data into reliable service-life predictions at the module level. Consequently, we identify the development of a standardized accelerated aging protocol for 2D material membranes as a critical task for the field. This protocol should encompass hydraulic pressure cycling, pH excursion testing, and oxidative cleaning tolerance under defined conditions, akin to the ASTM and ISO standards that underpin commercial polymeric membrane qualification [139,140].

### 7.4. Economic Feasibility and Life-Cycle Sustainability

Economic viability and environmental sustainability are prerequisites for industrial translation, yet both remain critically underaddressed in the graphene membrane literature. On manufacturing cost, commercial polyamide TFC membranes are produced at USD 5–15 m^−2^ [106], while CVD graphene currently costs USD 100–1000 m^−2^ at research scale [132,141], with techno-economic projections suggesting USD 20–50 m^−2^ at roll-to-roll volumes [38,141], approaching but not yet reaching commercial parity. GO-TFC membranes are considerably more cost-competitive at USD 15–40 m^−2^ at pilot scale [39,40], though economic viability ultimately depends on achieving service lifetimes of three to seven years that current stability data do not yet support. On energy consumption, the primary argument for graphene membranes is reduced operating pressure and lower specific energy relative to conventional RO (3–4 kWh m^−3^) [68,120]; however, this benefit is only realizable if salt rejection is maintained at lower transmembrane pressures, a condition current GO laminates do not consistently satisfy. The strongest near-term energy case, therefore, lies in nanofiltration, where operating pressures are already modest. On environmental impact, the Hummers method generates hazardous waste streams and carries a carbon footprint estimated at two to five times that of commercial polyamide membranes per unit area [142]; the Tour method reduces this to approximately 1.5–2.5 times, providing an environmental argument that complements its structural advantages [143]. We identify the development of standardized life-cycle assessment methodology for 2D material membranes as an urgent community priority.

### 7.5. Technology Readiness Assessment

Translating laboratory membrane performance into industrial deployment requires an honest assessment of where each application area currently stands. Table 6 maps the principal graphene-based membrane application domains to their estimated Technology Readiness Level (TRL), primary barriers to advancement, and indicative timelines to pilot-scale demonstration (TRL 6), providing a structured basis for prioritizing research investment and identifying the most tractable near-term opportunities.

Technology Readiness Levels in Table 6 follow the nine-level NASA/ESA framework adapted to membrane technology by Werber et al. [106]: TRL 1–3 (fundamental research to proof of concept), TRL 4–6 (laboratory to pilot-scale validation), TRL 7–9 (system demonstration to commercial deployment). Criteria applied: Water nanofiltration (TRL 4–5)—bench-scale permeance and rejection demonstrated reproducibly across multiple groups; best stability record is 240 h at 4 bar [86]; no pilot-scale module demonstration under continuous feed with representative fouling reported; stability data fall two orders of magnitude short of the ≥5000-h commercial qualification threshold. Gas separation, nanoporous graphene (TRL 2–3)—selectivity demonstrated at microscale only [110]; macroscale films achieve H_2_/CO_2_ selectivity of only 3–25 [92], reflecting unresolved pore size distribution and transfer-defect challenges. Osmotic energy harvesting (TRL 3–4)—power densities matching commercial PRO systems (1–5 W/m^2^) achieved under idealized laboratory conditions on small-area membranes only; no pilot-scale RED or PRO stack using GO ionic membranes has been reported. The lower TRL for osmotic energy harvesting versus water nanofiltration reflects: the more demanding simultaneous requirement for high ionic conductance AND high cation/anion selectivity; absence of hydraulic compaction stability data; and greater sensitivity of power output to concentration polarization losses at module scale.

**Table 6:** Technology Readiness Level (TRL) Assessment for Graphene-Based Membrane Applications.

Application	Current TRL	Best Demonstrated Performance	Primary Barrier to Next TRL	Timeline to TRL 6	Key References
Water nanofiltration (GO-TFC)	4–5	10–100 L m^−2^ h^−1^ bar^−1^; MWCO 300–1000 Da	Long-term hydraulic and chemical stability; module integration	3–5 years	[86,97,104]
Desalination/loose RO (GO)	3–4	>95% NaCl rejection at lab scale	Swelling control; defect-free large-area fabrication	7–10 years	[8,12]
Gas separation H_2_/CO_2_ (NPG)	2–3	H_2_/CO_2_ selectivity 3–25 at cm scale	Uniform nanopore distribution at m^2^ scale	10+ years	[92,110]
Gas separation CO_2_/N_2_ (GO)	3–4	CO_2_/N_2_ selectivity 20–40; 10^2^–10^3^ GPU	Humidity sensitivity; plasticization under mixed feeds	5–7 years	[113,114]
Organic solvent nanofiltration	3–4	Stable permeance in DMF, ethanol, acetone	Solvent-specific swelling; module sealing compatibility	5–7 years	[39,43]
Li^+^/Mg^2+^ ion recovery	3	Li^+^/Mg^2+^ selectivity ~10	Selectivity under real brine chemistry; fouling	7–10 years	[144]
Osmotic energy harvesting	3–4	~1–10 W m^−2^ at lab scale	Concentration polarization at module scale; cost per watt	7–10 years	[145,146,147]

Notes. TRL scale: 1 = basic principles observed; 3 = proof of concept; 4 = lab validation; 5 = relevant environment validation; 6 = pilot demonstration; 9 = full commercial deployment. All cited references appear in the manuscript reference list. Performance values represent best reported results under idealized laboratory conditions. NPG = nanoporous graphene; GO = graphene oxide; MWCO = molecular weight cut-off; GPU = gas permeation unit; DMF = dimethylformamide.

### 7.6. Emerging Directions: Beyond Graphene, Adjacent 2D Materials and Bio-Inspired Design

Several emerging directions hold particular promise for the field. Two-dimensional materials beyond graphene, including hexagonal boron nitride (hBN), transition metal dichalcogenides (MoS_2_, WS_2_), MXenes (Ti_3_C_2_T_x_), and covalent organic frameworks (COFs), offer complementary properties (chemical functionalization, intrinsic porosity, tunable hydrophilicity) that may overcome specific limitations of graphene and GO. Heterogeneous 2D laminates, combining two or more 2D materials in a designed sequence, represent a sophisticated route to membranes with properties unattainable by any single component. Artificial intelligence-guided membrane design, using machine learning models trained on experimental and simulation datasets to predict and optimize membrane composition and structure, is an accelerating frontier that promises to compress the design-fabricate-test cycle dramatically.

Bioinspired design principles, drawing inspiration from the exceptional selectivity and energy efficiency of biological ion channels, such as aquaporins, potassium channels, and chloride channels, continue to drive the development of novel membrane architectures. The integration of aquaporin proteins into graphene-supported bilayers, or the engineering of synthetic channels that replicate the structural characteristics of biological pores within graphene nanochannels, exemplifies a captivating convergence of synthetic nanomaterials and biomolecular engineering.

Table 7 presents a systematic performance comparison across the four material classes for the principal application domains covered in Section 6. Several conclusions emerge clearly. GO retains a decisive advantage in fabrication scalability, surface chemistry tunability, and cost, and remains the only 2D membrane material demonstrated at pilot scale. MXenes challenge GO most directly in water nanofiltration permeance, exceeding GO laminate values by one to two orders of magnitude (Table 7), and offer unique electrically switchable ion gating functionality, but oxidative instability limits operational lifetime to 100–500 h against GO’s >1000 h when properly cross-linked. COFs are the strongest competitive threat in gas separation and OSN, where geometric pore uniformity delivers H_2_/CO_2_ selectivities of 40–160 (Table 7) that GO’s polydisperse interlayer channels cannot match structurally. hBN is unchallenged in chemically aggressive environments, the only material in Table 7 stable across pH 1–14 and beyond 2000 h, where no other 2D membrane material operates reliably. GO membrane development is therefore most defensibly directed toward ion-selective nanofiltration, osmotic energy harvesting, and antifouling composites: the application spaces where GO’s tunability, scalability, and surface chemistry advantages are most consequential and least replicable by competing materials.

• Ti_3_C_2_T_x_ MXene ion sieving selectivity is electrically gated; value modulated by applied voltage.
• All experimental permeance values from bench-scale flat-membrane measurements under hydraulic pressure of 1–10 bar unless otherwise noted.
• Gas separation selectivities for GO and rGO represent mixed-gas measurements; COF and hBN values represent single-gas permeance ratios.
• Osmotic power densities measured under synthetic NaCl concentration gradients at patch scale; direct comparison with module-scale PRO data is not appropriate (see Section 6.4).
• Nanoporous graphene simulation values represent idealized defect-free pore arrays; experimentally demonstrated permeances and selectivities are substantially lower.
• N/D: not yet demonstrated at membrane scale. N/A: not applicable to this material class.

### 7.7. Environmental Impact and Sustainability of GO Membrane Production

The environmental sustainability of GO membrane technology has received limited systematic treatment in the graphene membrane literature. The classical Hummers method requires concentrated H_2_SO_4_, KMnO_4_, and H_2_O_2_, generating manganese-containing acidic waste streams requiring dedicated treatment. Estimates for analogous oxidative exfoliation processes suggest energy consumption of 50–200 kWh/kg GO at laboratory scale, substantially higher than the 5–20 kWh/kg estimated for conventional polyamide membrane casting. The Tour method eliminates NaNO_3_ and associated toxic NOx gas evolution, reducing hazardous byproduct generation while retaining comparable oxidation performance, and is therefore preferable from an environmental standpoint. Liquid-phase exfoliation in aqueous surfactant systems offers a lower-impact alternative but cannot yet achieve the oxidation level or interlayer tunability required for high-performance NF membranes. Membrane disposal and end-of-life: nanomaterial release during incineration or landfill disposal has not been systematically characterized for GO membrane modules. At elevated concentrations (>10 mg/L), GO nanosheets exhibit oxidative stress effects in aquatic organisms; the risk at environmentally realistic concentrations from membrane disposal remains unquantified. Conventional polyamide TFC membranes face their own end-of-life challenges (bisphenol A leaching from polysulfone supports, incomplete combustion byproducts), indicating that the relative environmental burden of GO versus polyamide disposal is not straightforwardly unfavorable for GO. The GO membranes could offer an indirect sustainability benefit if their higher permeance enables operation at lower transmembrane pressure, reducing energy consumption per unit permeate volume, but only if GO membranes achieve commercial lifetimes (5–7 years), which current stability data (best: 240 h) do not support. A full life cycle assessment comparing GO versus conventional polyamide NF membranes is not yet available and represents an important research need accompanying any pilot-scale demonstration.

## 8. Conclusions

This review has moved beyond material cataloguing to extract mechanistic design rules and identify the specific questions that remain genuinely open. On transport mechanism, the evidence assembled here supports a three-regime model, viscous near-frictionless flow through pristine graphitic corridors, activated hopping across oxygenated constriction sites, and solution–diffusion as the overarching thermodynamic driving force, with sufficient independent experimental and computational backing to treat it as an operational design framework rather than a hypothesis. The steric-only interpretation of the Joshi et al. ionic cut-off is superseded: partial dehydration energy barriers, Donnan exclusion from edge carboxylates, and C/O-dependent d-spacing variation each contribute to apparent sieving, and their weights shift with oxidation state, pH, and ionic strength. What remains unresolved is the quantitative partitioning between regimes across the C/O ratio space, and a unified rejection equation integrating all three exclusion contributions has yet to be validated experimentally.

On computation, the parity analysis in Section 3.3 establishes that classical MD overpredicts GO laminate permeance by 3–8×, correctable by a tortuosity–porosity factor derived from wet-state XRD, and that GO-specific machine-learning force fields (GAP, MACE) reduce this discrepancy to within ×1.5–2. Nuclear quantum effects are most consequential for proton transport applications; for ion rejection, classical MD remains adequate within typical force field uncertainties. For water permeance, published bulk PIMD studies indicate NQEs increase diffusivity by approximately 15–50% relative to classical MD [83,85], suggesting they may contribute to the residual MD–experiment discrepancy not accounted for by the tortuosity–porosity correction alone, but their magnitude in GO laminate geometry is unquantified and constitutes a priority target for future path-integral simulation.

Five design recommendations follow directly from this analysis. First, C/O ratio and epoxy cross-link density should be co-optimized rather than adjusted independently, targeting a wet-state d-spacing of 0.65–0.72 nm for divalent exclusion, with wet-state XRD at operating ionic strength as the mandatory characterization metric. Second, for GO-TFC membranes below 50 nm selective-layer thickness, support resistance accounts for 30–60% of total hydraulic resistance and must be minimized through pore architecture optimization; ignoring it systematically overestimates intrinsic GO permeance. Third, covalent interfacial bonding between the GO layer and the polymeric support is necessary for sustained operation above 5 bar; van der Waals adhesion alone is insufficient under pressure cycling. Fourth, zwitterionic functionalization is the preferred antifouling strategy, delivering >95% flux recovery after hydraulic cleaning and avoiding the Ag^+^ leaching and progressive efficacy loss associated with silver nanoparticle incorporation. Fifth, in electrically gated rGO membranes, applied voltage must be calibrated against pH-dependent surface charge density, since the Donnan coefficient is exponentially sensitive to the product z·F·Δψ_D/RT and small pH shifts near the carboxylate pK_a_ (~4–5) can negate the electrostatic bias entirely.

Three experimental advances would most decisively move the field forward. The standardized 500-h hydraulic stability protocol proposed in Section 7.2, 10 bar, 0.2 M NaCl, pH 6–8, 25 °C, must be executed across multiple independent laboratories on chemically equivalent cross-linked GO membranes; without it, durability claims remain non-comparable, and TRL advancement for water nanofiltration is not credible. A systematic multi-laboratory parity study, reporting classical MD and ML force field permeance predictions alongside experimental values for membranes spanning C/O ratios of 1.4–2.6, would simultaneously validate the three-regime framework quantitatively and establish ML force fields as the community simulation standard. Finally, the first continuous module-scale demonstration, spiral-wound or hollow-fiber format, operated for ≥1000 h under realistic feed and cleaning conditions, remains the single largest gap between the current TRL 4–5 status of GO nanofiltration and the pilot-scale validation required to attract industrial commitment. Progress on these three fronts, in parallel with ML force field development and mechanistic refinement, would place graphene-based membrane technology on a credible trajectory toward deployment in water treatment, gas separation, and osmotic energy harvesting.

## Figures and Tables

**Table 2:** MD-Predicted vs. Experimentally Measured Water Permeance for Graphene-Based Membranes. Systematic comparison across C/O ratio, d-spacing, and simulation method.

Membrane Type	C/O Ratio	d-Spacing (nm)	MD-Predicted Permeance (L m^−2^ h^−1^ bar^−1^)	Experimental Permeance (L m^−2^ h^−1^ bar^−1^)	MD/Exp Ratio	Simulation Method	Key References
GO laminate (Hummers)	1.4	0.98	180–240	28–45	5.3–6.7	Classical MD (SPC/E)	[9,74]
GO laminate (Tour)	2.2	0.82	95–130	22–38	3.1–4.8	Classical MD (SPC/E)	[12]
GO cross-linked (epoxy)	2.1	0.72	60–85	18–30	2.8–3.5	Classical MD (SPC/E)	[12]
rGO laminate	3.8	0.65	40–55	12–22	2.5–3.2	Classical MD (TIP4P)	[39,86]
GO laminate (Hummers)	1.5	0.90	140–180	30–52	1.6–2.1 *	ML force field (GAP)	[87]
GO laminate (Tour)	2.3	0.80	55–75	28–40	1.6–2.1 *	ML force field (MACE)	[81,82]
Nanoporous graphene (pore Ø ~0.45 nm)	—	—	1200–2800	400–900	2.1–4.2	Classical MD (SPC/E)	[41,88]

Notes. Permeance values represent ranges reported across independent studies using membranes of nominally equivalent C/O ratio and d-spacing. MD/Exp ratio > 1 indicates model overprediction; * ML force-field predictions fall within the ×2 parity band. All experimental values are from flat-membrane, bench-scale measurements under hydraulic pressure of 1–10 bar. Abbreviations: rGO, reduced graphene oxide; GAP, Gaussian approximation potential; MACE, multi-ACE architecture; TIP4P, transferable intermolecular potential 4-point; SPC/E, extended simple point charge model; d-spacing measured by XRD in hydrated state.

**Table 3:** Recommended Tiered Simulation Workflow for GO Membrane Transport Studies.

Simulation Tier	When to Use	Design Role
Classical MD (SPC/E, TIP4P, OPLS-AA)	Initial screening: parameter-space exploration across C/O ratios and d-spacings	Rank-order membrane compositions; identify qualitative permeance trends; treat all flux values as upper bounds and calibrate against wet-state XRD tortuosity correction (τ^2^/ε)
ML-FF (GAP, MACE)	Mechanistic validation where classical MD shows demonstrable bias; quantitative regime partitioning studies	Resolve viscous-flow vs. activated-hopping contributions across C/O ratio space; correct systematic ion free-energy barrier errors; target within ×1.5–2 of experimental permeance
Path-Integral Methods (PIMD, TRPMD)	Proton or light-ion transport; osmotic energy harvesting membranes; proton exchange membrane applications	Capture NQE contributions to proton mobility; correct the 2–4× classical MD underestimation of proton conductance; mandatory for any application where tunnelling through hydrogen-bond networks is a rate-limiting step

Abbreviations: MD, molecular dynamics; ML-FF, machine-learning force field; GAP, Gaussian approximation potential; MACE, multi-ACE architecture; PIMD, path-integral molecular dynamics; TRPMD, thermostatted ring-polymer molecular dynamics; NQE, nuclear quantum effect; C/O, carbon-to-oxygen ratio; τ, tortuosity; ε, accessible porosity fraction.

**Table 4:** Parameter identification protocol for HP/ENP continuum modeling of GO laminate membranes.

Parameter	Symbol	Measurement Method	Typical Range (GO Laminates)
Channel height	*h* (nm)	Wet-state XRD (002); subtract 0.34 nm for GO sheet thickness	0.31–0.84 nm
Tortuosity–porosity factor	*t*2/*e*	XRD FWHM via Scherrer equation + BET accessible porosity	4–36
Surface charge density	*s* (mC/m^2^)	Streaming potential at operating pH and ionic strength; Equation (3)	−5 to −60 mC/m^2^
Confined water viscosity	*h* (mPa s)	Bulk (0.89 mPa s) × correction 1.5–3× for h < 0.8 nm	0.9–2.7 mPa s
Effective ion diffusivity	*Di*,*eff* (m^2^/s)	PFG-NMR on swollen GO powder, or bulk Di/t2	10–30% of bulk
Steric partition coefficient	*Ks*,*i*	Geometric; Equation (2) using hydrated ionic radius of species i	0.01–0.85

**Table 5:** Hydraulic Stability Data for GO Membranes Reported in the Primary Literature, with Proposed Standardized Benchmark Protocol.

Reference	Cross-Linking Strategy	Pressure (bar)	Duration (h)	Flux Decline (%)	Feed Chemistry	Notes
[86]	Epoxy resin (bisphenol A)	4 bar	240 h	**~8%**	0.1 M NaCl aq. solution	Best documented hydraulic stability record to date
[12]	Epoxy (varied loading)	1–5 bar	72 h	**12–18%**	DI water; 0.5 M KCl	d-spacing tuned 0.65–0.98 nm; no long-term data
[39]	None (rGO; thermal reduction)	2 bar	120 h	**~22%**	DI water	Reduction temp. 220 °C; swelling not fully suppressed
[136]	Sodium tetraborate (borate, inorganic)	1 bar	24 h	**~30%**	DI water, pH 7	Reversible B–O–C cross-linking; bond hydrolysis causes instability at pH < 5
[137]	glutaraldehyde (GA)	5 bar	3 h	**~14%**	MgCl_2_ tested	Short compaction-phase flux decline at 5 bar; no long-duration (48 h) data reported.
[9]	None (pristine GO)	N/A (vapor)	—	N/A	Water vapor (humidity cycling)	No hydraulic pressure data; vapor only
[74]	TDI (diisocyanate)	5 bar	100 h	**~20%**	0.2 M NaCl	Organic cross- linker; potential toxicity concern
Standardized protocol (proposed)	To be specified	10 bar (target)	500 h (target)	**<10% (target)**	0.2 M NaCl; pH 6–8; 25 °C	Proposed minimum benchmark; see Section 7.2

Notes. Flux decline is reported as percentage decrease in permeance from initial value at end of test period. ‘N/A’ indicates no hydraulic pressure stability data reported. TDI, toluene diisocyanate; GA, glutaraldehyde; rGO, reduced graphene oxide; DI, deionized. Proposed protocol represents the minimum conditions recommended for standardized accelerated aging tests; see Section 7.2 for full rationale. All data from flat-membrane bench-scale studies; no module-level stability data are currently available in the open literature for GO membranes.

**Table 7:** Comparative Performance of Emerging 2D Membrane Materials Across Key Application Domains.

Material	Water NF Permeance (L m^−2^ h^−1^ bar^−1^)	NaCl Rejection (%)	Gas Sep. H_2_/CO_2_ Selectivity	OSN Permeance (L m^−2^ h^−1^ bar^−1^)	Ion Sieving Na^+^/Mg^2+^ Selectivity	Osmotic Power (W m^−2^)	Operational Stability	Max. Operating pH	Scalability	References
GO laminate	10–100	85–98	5–20	5–40	2–8	1–10	>1000 h (cross-linked)	3–10	Pilot scale (90 × 30 cm)	[9,12]
rGO laminate	50–300	70–92	10–35	10–60	1.5–4	2–8	500–800 h	3–10	Lab–pilot	[39,86]
Ti_3_C_2_T_x_ MXene	1000–4000	80–95	3–12	20–120	3–10*	3–15	100–500 h (oxidation-limited)	4–9	Lab scale (<100 cm^2^)	[116,148]
COF (imine/triazine)	50–300	88–97	40–160	30–200	4–12	N/D	>500 h (dry) <200 h (aq.)	4–9	Lab scale (<10 cm^2^)	[116,149]
hBN laminate	8–40	85–97	15–50	5–25	2–6	N/D	>2000 h	1–14	Lab scale (<50 cm^2^)	[150]
Nanoporous graphene	10^3^–10^5^ (simulated)	99+ (simulated)	10^2^–10^4^ (simulated)	N/D	>100 (simulated)	N/D	Limited exp. data	N/A	Sub-cm^2^ (exp.)	[41,42]

Notes. Color coding: **Green** = best-in-class performance; **Amber** = moderate/competitive; **Red** = limited or inferior; Grey = not yet demonstrated (N/D) or not applicable (N/A).

## Data Availability

No new data were created or analyzed in this study. Data sharing is not applicable to this article.
